# Radiochemistry on electrodes: Synthesis of an ^18^F-labelled and *in vivo* stable COX-2 inhibitor

**DOI:** 10.1371/journal.pone.0176606

**Published:** 2017-05-02

**Authors:** Artem Lebedev, Jing Jiao, Jason Lee, Fan Yang, Nathanael Allison, Harvey Herschman, Saman Sadeghi

**Affiliations:** 1 UCLA Department of Molecular and Medical Pharmacology, David Geffen School of Medicine, Los Angeles, California, United States of America; 2 Traceability, Culver City, California, United States of America; 3 UCLA Crump Institute for Molecular Imaging, Los Angeles, California, United States of America; 4 The Department of Chemistry at The Scripps Research Institute, La Jolla, California, United States of America; 5 UCLA Biomedical Physics Interdepartmental Program, Los Angeles, California, United States of America; 6 UCLA Ahmanson Translational Imagining Division, Los Angeles, California, United States of America; Biomedical Research Foundation, UNITED STATES

## Abstract

New radiochemistry techniques can yield novel PET tracers for COX-2 and address the shortcomings in in vivo stability and specificity, which have held back clinical translation of tracers to image COX-2 expression. Current techniques limit radiosynthesis to analogs of the COX-2 inhibitors with fluorine-18 added via a carbon chain, or on an aromatic position which renders the radiolabeled analog less specific towards COX-2, resulting in tracers with low *in vivo* stability or specificity. To solve this problem, we have developed a new high affinity, ^18^F-labelled COX-2 inhibitor that is radiolabeled directly on a heteroaromatic ring. This molecule exhibits favorable biodistribution and increased metabolic stability. Synthesis of this molecule cannot be achieved by traditional means; consequently, we have developed an automated electrochemical radiosynthesis platform to synthesize up to 5 mCi of radiochemically pure ^18^F-COX-2ib in 4 hours (2% decay-corrected radiochemical yield). *In vitro* studies demonstrated clear correlation between COX-2 expression and uptake of the tracer. PET imaging of healthy animals confirmed that the molecule is excreted from blood within an hour, mainly through the hepatobiliary excretion pathway. *In vivo* metabolism data demonstrated that > 95% of the injected radioactivity remains in the form of the parent molecule 1 hour after injection.

## Introduction

There is strong evidence suggesting a relationship between inflammation and carcinogenesis, as well as neuroinflammation and CNS disease progression. Several processes that are involved in carcinogenesis, including apoptosis, angiogenesis, cell proliferation, invasiveness and metastasis, are correlated with COX-2 overexpression. Epidemiological data support the correlation of COX-2 overexpression with cancer, since aspirin or other NSAIDs lower incidence of deaths from various types of cancer[1]. Genetic studies have provided further correlation between carcinogenesis and COX-2 overexpression. For example targeted COX-2 deletion led to decreased intestinal polyps in female mice [2] and enhanced COX-2 expression is sufficient to induce mammary gland tumorigenesis[3]. COX-2 expression has also been shown to have a direct role in modulating breast cancer progression [4].

Cyclooxygenase-2 (COX-2), located on the luminal side of the endoplasmic reticulum and nuclear membrane, plays a major role in regulating the rate of conversion of arachidonic acid to the various prostanoids and their downstream products[5]. COX-2 overexpression is a characteristic feature of many premalignant neoplasms[6] and appears to be both a marker and an effector of neural damage, both after a variety of acquired brain injuries and in natural or pathological aging of the brain[7]. While co-expression of COX-2 with tumor metastatic phenotype has been observed in certain types of cancer[8], evidence of a direct role for COX-2 in carcinogenesis and neurodegenerative processes remains controversial, and, in the absence of a viable COX-2 *in vivo* imaging agent, hypotheses either way cannot be confirmed without the availability for non-invasive longitudinal studies. Developing a non-invasive COX-2 imaging agent will be of great value, contributing to our understanding of the molecular mechanisms associated with inflammatory processes, by monitoring COX-2 levels throughout the progression of diseases such as neurodegenerative Alzheimer disease and Parkinson’s disease. Furthermore, early detection of this inflammation related process, i.e., induction of COX-2 expression, can potentially stratify patients and provide a rationale for selective therapies and their optimization in treatment of CNS disorders and cancers. One such example is a clinical study demonstrating stratification of patients with the presence of COX-2 in premalignant cancer lesions as an important determinant of their response to adjuvant celecoxib therapy [9]. Currently, only *ex vivo* analysis can provide quantitative information on COX-2 expression. However, *ex vivo* analysis is laborious, will not provide localization and biodistribution, and can be inaccurate, since COX-2 mRNA and protein are not stable *ex vivo* [10].

Positron emission tomography (PET) is a real-time, *in vivo* three dimensional imaging technique that has unparalleled specificity and sensitivity for visualizing biochemical processes[11]. It is uniquely suited to provide data on *in vivo* expression of COX-2 and its involvement in disease development and progression. This modality is already widely used in the clinic and clinical translation of novel PET tracers has recently yielded an array of newly approved tracers. [12]. The unparalleled sensitivity of this method makes it the only viable candidate for visualization of low abundance targets, such as COX-2. PET relies on the administration of an exogenous tracer–a radiolabeled molecule with a known biodistribution, administered at sub-pharmacological levels for visualizing and quantifying molecular processes *in vivo*. While a variety of positron-emitting radionuclides can be used for this purpose, ^18^F remains the staple for clinical PET tracers due to the favorable 109.8 min radioactive half-life, 97% low energy positron emission and the fact that addition of a small fluorine atom minimally perturbs the parent molecule. Desirable properties of ^18^F label have given rise to a whole research field of ^18^F radiolabeling and continuous advances in this field have yielded an impressive set of tools for labeling of a wide range of molecules.[13]

Despite these advances, a clinical translatable tracer for visualization of COX-2 expression through PET imaging still awaits the development of an adequate probe. A large set of potent COX-2 inhibitors have been developed for therapeutic applications; these molecules can potentially be used as vectors for radioactive labels. However, low tolerance of these molecules to structural modifications has left very few options for labeling using traditional methodologies. Most known attempts have yielded tracers suffering from either low metabolic stability or low affinity to the enzyme. Previous efforts to address this problem are summarized in a recent review.[14] However, despite attracting substantial attention, finding a suitable COX-2 PET tracer remains an unresolved challenge.

**Fig 1:**
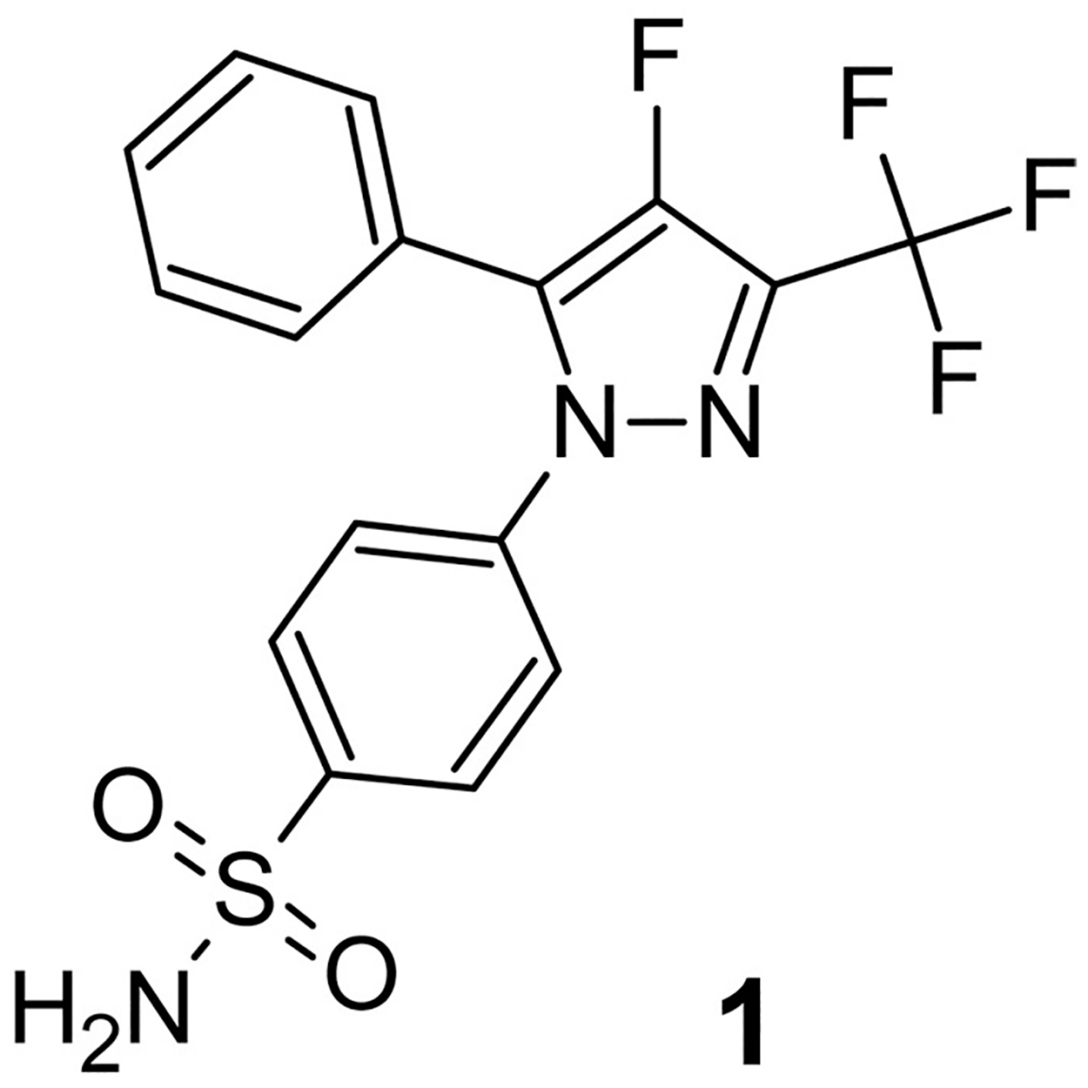
Celecoxib analog 1. IC_50_ = 1.7 nM.

Among COX-2 inhibitors reported to date, the celecoxib analog **1**[15] (Fig 1) stands out because of two major advantages for PET imaging: 1) low nanomolar affinity to the enzyme and 2) presence of a fluorine atom directly attached to heteroaromatic moiety. Radiolabeling of this molecule can be performed, with no structural perturbation, by replacing the natural fluorine isotope with ^18^F-fluorine. The introduced radioactive label will be present at a metabolically stable position. The importance of minimal structural perturbation is highlighted by extensive structure–activity studies that suggest bulky substituents on COX-2 inhibitors are not tolerated *in vivo*.[15] Tracers having a fluorine atom attached to the aromatic ring generally exhibit better metabolic stability compared to agents with the ^18^F label attached to aliphatic chain, which often experience rapid defluorination, especially if excreted through hepatobiliary pathway.[16][17]. Working within the confines of traditional radiochemistry, several groups have attempted to place an ^18^F-fluorine on an aromatic moiety of various analogs of COX-2 inhibitors. Unfortunately, traditional aromatic radiolabeling methods are restricted in the placement of the ^18^F label to only a few positions on the COX-2 inhibitor scaffold. However, substituents on the aromatic ring alter the binding affinity of the radiolabeled COX-2 inhibitors. Such compounds may show probe uptake in COX-2–expressing cells, but were ineffective *in vivo* [18–20]. Placing the ^18^F-fluorine label on the five membered ring thus emerges as an attractive approach, but there are very few examples of either late stage “cold” 19F-fluorination or ^18^F radiolabeling of electron rich five-membered heteroaromatic rings.[21],[22]

Radiofluorination of aromatic molecules in general has so far remained a highly desirable but elusive goal. Unfortunately, ^18^F-fluoride, the most accessible form of radioactive fluorine, exhibits low reactivity in classic nucleophilic substitution. Historically, electrophilic reactions involving ^18^F-F_2_ gas have been a solution for this problem, but these reactions suffer from low specific activity, lack of availability of ^18^F-F_2_ and have experimental challenges associated with the handling of corrosive F_2_ gas. Precursors bearing a strongly electron withdrawing group can react with fluoride directly, but this method severely restricts the scope of target tracers [23,24]. A number of methods have been developed to address these challenges. These methods focus on purely chemical methodologies, employing various catalysts,[25] organometallic[26] and iodonium[27] leaving groups or strong oxidizing reagents[28], to facilitate unfavorable interaction between the negatively charged fluoride anion and the electron rich aromatic moiety. Recent reviews have summarized these approaches.[29,30]

Electrochemical fluorination stands out from the late stage radiofluorination methods described above.[31],[32] This method relies on the oxidation of the precursor on an electrode surface maintained at a potential above the oxidation potential of the precursor, to generate a cationic species reactive toward fluoride anion. In the context of selective fluorination of organic molecules, this method has been well studied and applied to a variety of molecules.[33] However, utilization of this methodology in fluorination of aromatics and radiosynthesis has been lagging.

## Materials and methods

All animal experiments were performed according to protocol approved by the UCLA Office of Animal Research Oversight. Isoflurane anesthesia was used for the invasive procedures, animals were sacrificed by carbon dioxide asphyxiation.

Electrochemical radiolabeling was performed using an in-house built radioelectrochemical synthesizer. A detailed description and evaluation of the apparatus is under preparation for publication. Fluidic schematic of the platform can be found in Supporting information (S2 Protocol, Fig 1). No-carrier added [^18^F]fluoride was produced via ^18^O(p,n)^18^F nuclear reaction in a RDS-112 (Siemens, USA) cyclotron by 11 MeV proton bombardment of 95% oxygen-18 enriched water ([^18^O]H_2_O, Rotem, Inc., Israel) in a tantalum target with a beam strike volume of 860μL. A beam current of 35 μA for 60 min yielded ~1100 mCi of [^18^F]fluoride, which was directly delivered to the ^18^F vial of the radioelectrochemical synthesizer.

After delivery of radioactivity from the cyclotron, [^18^F] fluoride in target water was pushed through a cartridge containing 10 mg of anion exchange resin (Bio-Rad AG MP-1M), followed by 6 ml of dry acetonitrile. The cartridge was further dried by blowing dry nitrogen at 10 psi for 5 min.

After the cartridge was dried, 4 ml of solution of precursor (50 mM), Bu_4_NClO_4_ (50 mM) and Et_4_NF*4HF (20 mM) in dry acetonitrile was pushed through the MP-1 cartridge (controlled flow rate of 40 uL/s) and directed into the cylindrical Teflon reactor. The use of the fluoride salt is primarily dictated by the need for an electrochemically inert anion capable of replacing fluoride on anion exchange cartridge. While this limits the achievable specific activity, the added carrier fluoride is not a fundamental prerequisite for the electrochemical reaction. Details on the synthesis of precursors and standards can be found in Supporting information (S1 Protocol).

The solution was then electrolyzed at ambient temperature using Ø1.0 mm Pt wire electrodes (working electrode ~800 mm^2^, auxiliary electrode ~1300 mm^2^) and Ø1.5 mm Ag wire as a pseudo reference electrode. Electrolysis and concurrent fluorination in the cell was conducted for 70 min in 700 cycles using an Autolab PGSTAT204 driven by Nova 1.9 software (Metrohm USA). Each cycle consisted of 5 sec of working phase at 2.7 V and 1 sec of recovery phase at 0.3 V. A controlled stream of dry nitrogen (0.5 to 2 ml/min) was blown across the surface of the solution to remove hydrogen gas formed at the auxiliary electrode. Due to incomplete conversion of the precursor, chemical yield of the reaction can be improved by extending the electrolysis process, although this will not improve radiochemical yield because of the competing radioactive decay.

After hydrolysis, the dark yellow reaction mixture was transferred into a pre-heated reactor charged with 1 ml of 37% HCl_aq_. The mixture was stirred for 15 min and 12 ml of water was added. The resulting slurry was passed through a C-18 SPE cartridge (Waters Sep-Pak Classic Short). The product adsorbed on the cartridge was washed off with 1 ml of EtOH followed with 1 ml of water.

The resulting suspension of crude product in 50% EtOH was transferred into the loading loop of the prep-HPLC part of the system and purified using a Phenomenex Gemini column (21x250 mm) at 20 ml/min of 47% MeCN in water as an eluent. The radioactive peak at approx. 30 min was collected in approx. 20 ml of eluent.

20 ml of water was added to the collected fraction and the solution was passed through a C-18 SPE cartridge (Waters Sep-Pak Light). The product was washed off the cartridge using 400 uL of EtOH followed by 400 uL of water.

**Fig 2:**
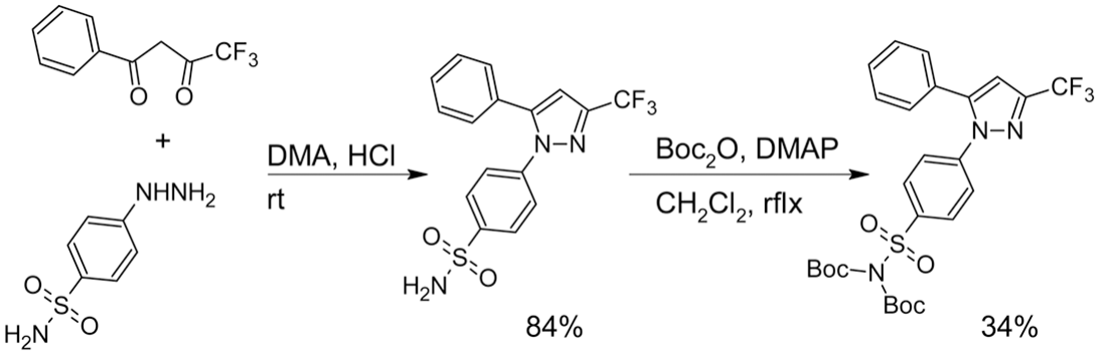
Synthesis of radiolabeling precursor.

After radioactive decay and losses in the system, typically, 2 to 5 mCi of the radiochemically pure final product was obtained. The entire process takes approx. 4 hours (0.8 to 2% DCY). ^1^H, ^13^C, ^19^F NMR, ESI MS as well as analytical HPLC of non-radioactive carrier ^**19**^**F-1** and radioactive ^**18**^**F-1** obtained in this reaction are provided in the supporting information (S1 Spectra). Specific activity at the end of synthesis was approximately 3 Ci/mmol. (S2 Protocol, Fig 2). This is in line with typical carrier added syntheses and approximately 1000 times lower than a typical no-carrier added synthesis.

Detailed protocols for in-vitro cell-association assay (S3 Protocol), *in vivo* imaging (S4 Protocol) and in-vivo metabolism studies (S5 Protocol) can also be found in supporting information.

## Results

### Radioelectrochemical synthesis

Here we present a new method of radiofluorination of the pyrazole moiety based on electrochemical oxidation. We demonstrate the method by synthesis of an isotopomer of **1**. The resulting molecule ^18^F-**1**, shows both *in vivo* stability not previously demonstrated for ^18^F-labelled COX-2 inhibitors and *in vitro* specificity to COX-2 expression, making it a promising candidate as a molecular probe for visualization of COX-2 expression. We also demonstrate short blood half-life and fast excretion in a healthy mouse model. In its current form, the method only yields a marginal specific activity of 3 Ci/mmol (S2 Protocol, Fig 2), which needs to be improved before the tracer can be used as a sensitive *in vivo* imaging agent for COX-2 expression.

**Fig 3:**
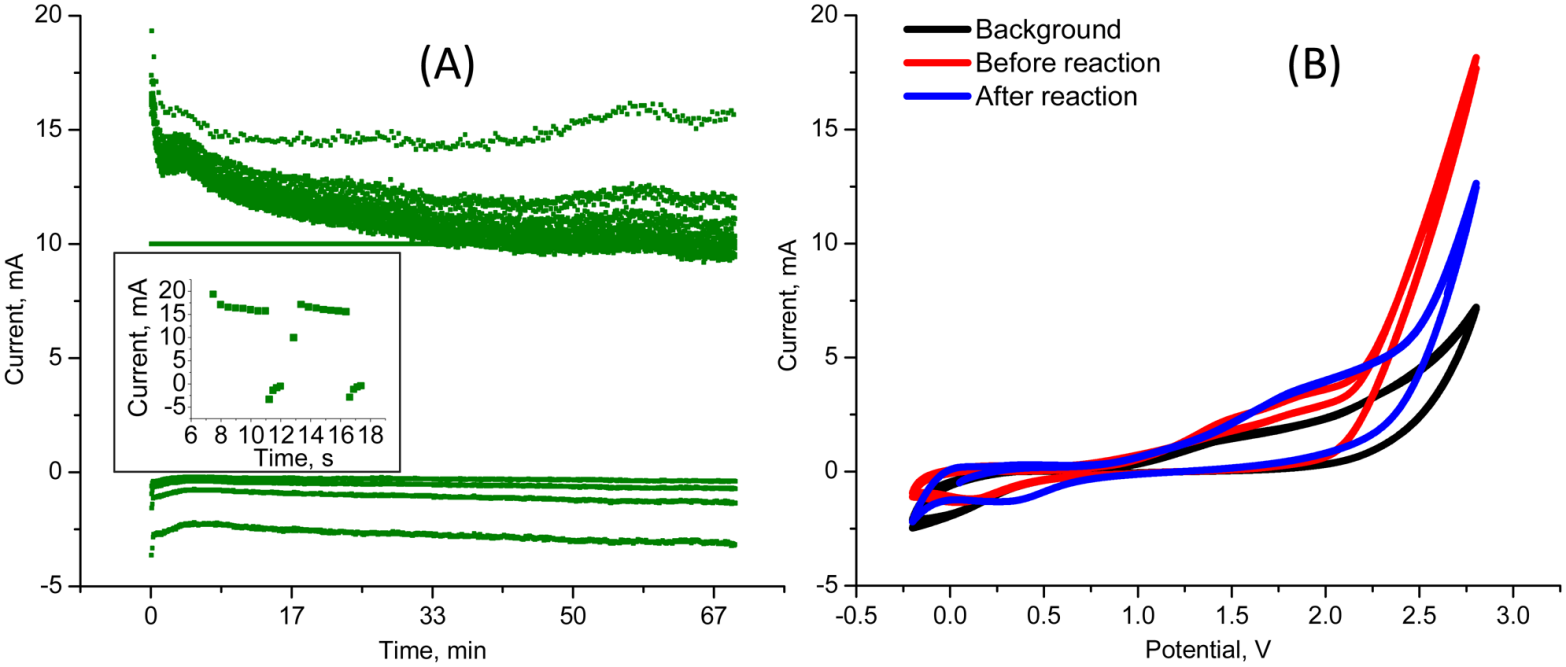
Electrochemical characteristics of radiolabeling process. A) is a typical current vs time plot for the duration of electrolysis and B) shows the cyclic voltammogram of the background and the reaction mixture before and after electrolysis.

**Fig 4:**
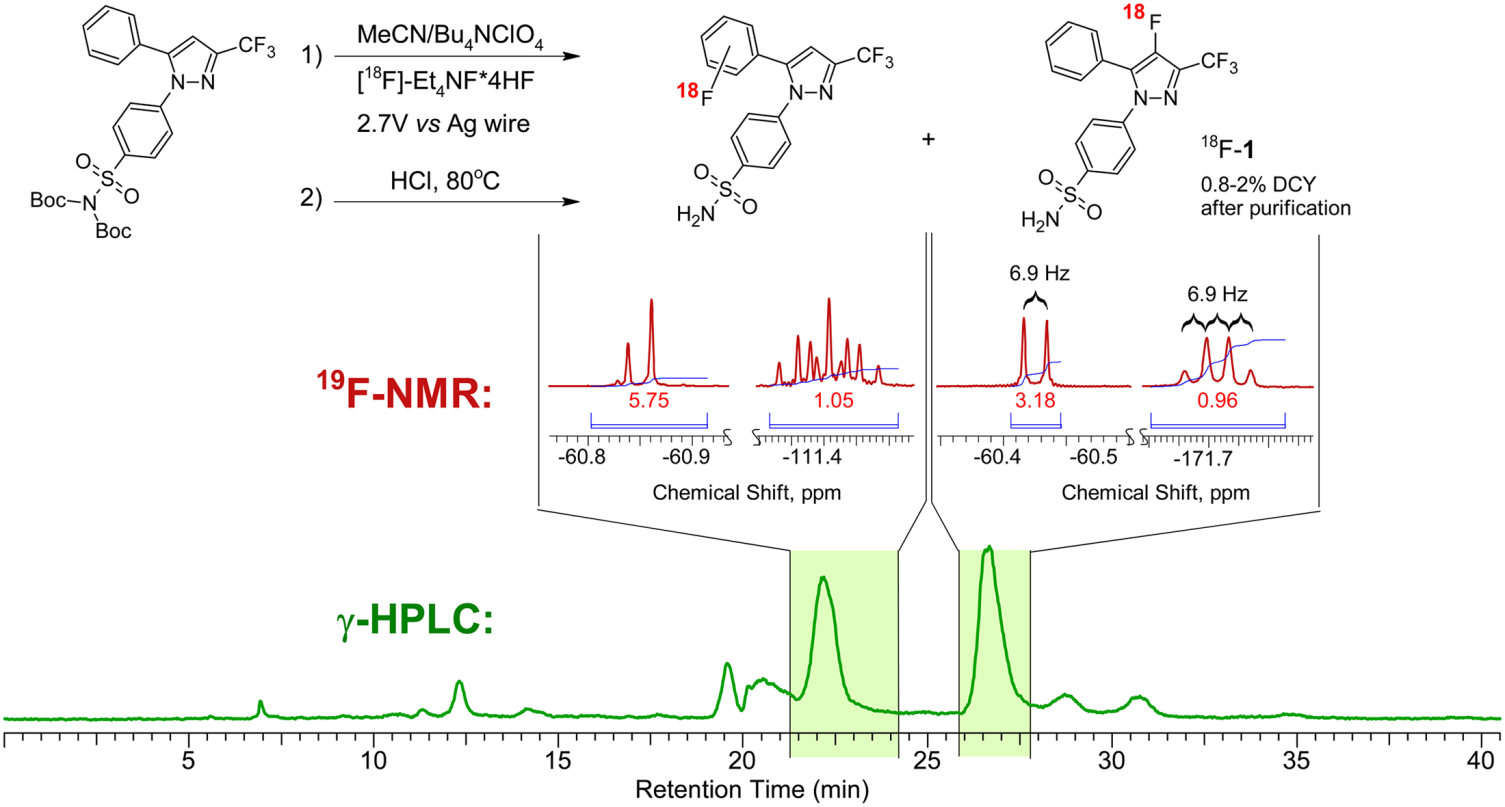
The reaction scheme of the electrochemical radiosynthesis of COX-2 inhibitor ^18^F-1. Green trace is the preparative radiochromatogram of the reaction mixture. Areas shaded green correspond to the collected fractions, the key areas of the ^19^F-NMR spectra of the collected fractions are shown above.

We prepared ^**18**^**F-1**, a radiolabeled version of COX-2 inhibitor **1**, using the electrochemical radiofluorination approach presented in Fig 4. The final precursor for the electrochemical radiolabeling was synthesized via a two-step process presented in Fig 2. Electrolysis was performed under pulsating potentiostatic conditions on platinum wire electrodes. Oscillating potential on the working electrode was used to prevent electrode surface fouling. Potential was kept at 2.7 V for 4 seconds, followed by a 1 sec 0.3 V pulse to regenerate the surface. Fig 3a shows a typical current vs time curve for this experiment. Inset in the figure demonstrates the section of the curve that corresponds to two consecutive voltage oscillations. Fig 3b represents cyclic voltammograms (CV) of the reaction mixture before and after electrolysis, along with the CV of the background solution (electrolyte + Et_4_NF*4HF), which guided the selection of the oxidation potential for the reaction. Onset potential for the oxidation of the precursor was ~2.2 V and at the 2.7 V operating voltage of the cell, ~10 mA of current was due to precursor oxidation above the background current at this potential (Fig 3b). As expected, after electrolysis, oxidation current was found to be lower due to both precursor and electrolyte consumption.

The structure of the final product was confirmed using NMR and AP-ESI mass spectroscopy. Due to the carrier added mode of the synthesis, the amount of the product produced during radiosynthesis was sufficient for NMR characterization; therefore, all analysis was done on the decayed samples obtained in the radiolabeling runs. Typically, semipreparative HPLC trace (Fig 4, green trace) of the reaction mixture contains two major radioactive peaks along with smaller unidentified byproducts. Both compounds were isolated and analyzed using ^1^H, ^13^C, ^19^F NMR. ^19^F NMR (Fig 4, red trace) clearly shows that the compounds of the earlier fraction do not contain any ^19^F nuclei coupled with -CF_3_ group (singlets at ~-60.9 ppm) while the compound in the later fraction exhibits a spectral pattern consistent with–F substituent (q, 6.9 Hz, -171.7 ppm) coupled with -CF_3_ group (d, 6.9 Hz, -60.4 ppm). ^1^H and ^13^C NMR as well as AP-ESI mass spectra are also consistent with the structure of 1 (S1 Spectra).

Up to 5 mCi of the final purified product was obtained using this procedure (2% DCY). After HPLC purification and subsequent reformulation, chemically and radiochemically pure doses of ^18^F-**1** were produced and used in subsequent studies. Analytical HPLC traces of the pure injectable dose produced following this procedure is presented in the supporting information (S1 Spectra). Due to low solubility in aqueous ethanol, the compound was formulated in 40% EtOH prior to *in vitro* or *in vivo* administration.

### *In vitro* cell uptake studies

To demonstrate the potential of ^**18**^**F-1** for quantification of COX-2 expression, *in vitro* COX-2 dependent uptake of the probe in LPS-treated RAW264.7 mactrophage-like cells was demonstrated. Further details on the *in vitro* uptake experiments can be found in the supporting information (S3 Protocol).

**Fig 5:**
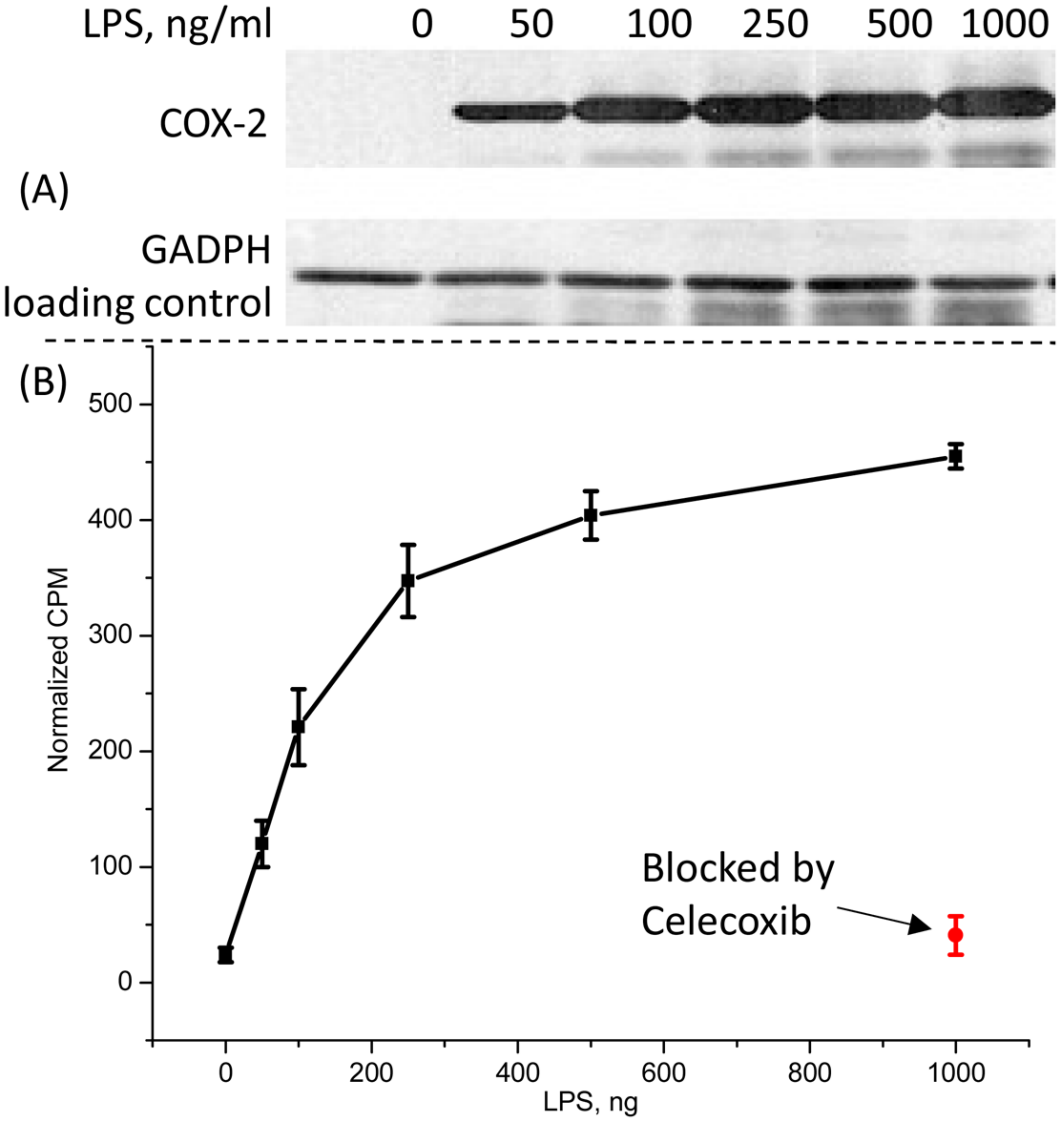
In vitro experiments. (A) Western blot analysis of LPS treated cells. The upper panel is COX-2 expression and the lower blot is GAPDH loading control, (B) In vitro COX-2 dependent uptake of the probe in LPS-treated RAW264.7 macrophage-like cells, normalized to 1000 ng/ml of LPS.

A clear dependence of the ^18^F-1 uptake on the concentration of LPS was observed in these experiments. Fig 5 shows progressive increase in the uptake with increasing LPS concentration and COX-2 levels up to 400 ng/ml, followed by uptake saturation beyond LPS concentrations exceeding 500 ng/ml. Analysis of the western blot data overlaid in Fig 5 illustrates that COX-2 expression with LPS stimulation also starts to saturate above 400ng/ml and probe uptake and expression data closely parallel one another.

Additionally, LPS induced uptake of ^**18**^**F-1** was blocked by 32 μg/ml of Celecoxib, a selective COX-2 inhibitor (Fig 5), supporting the conclusion that COX-2 expression is responsible for probe retention in the cells.

### *In vivo* metabolism and PET imaging

**Fig 6:**
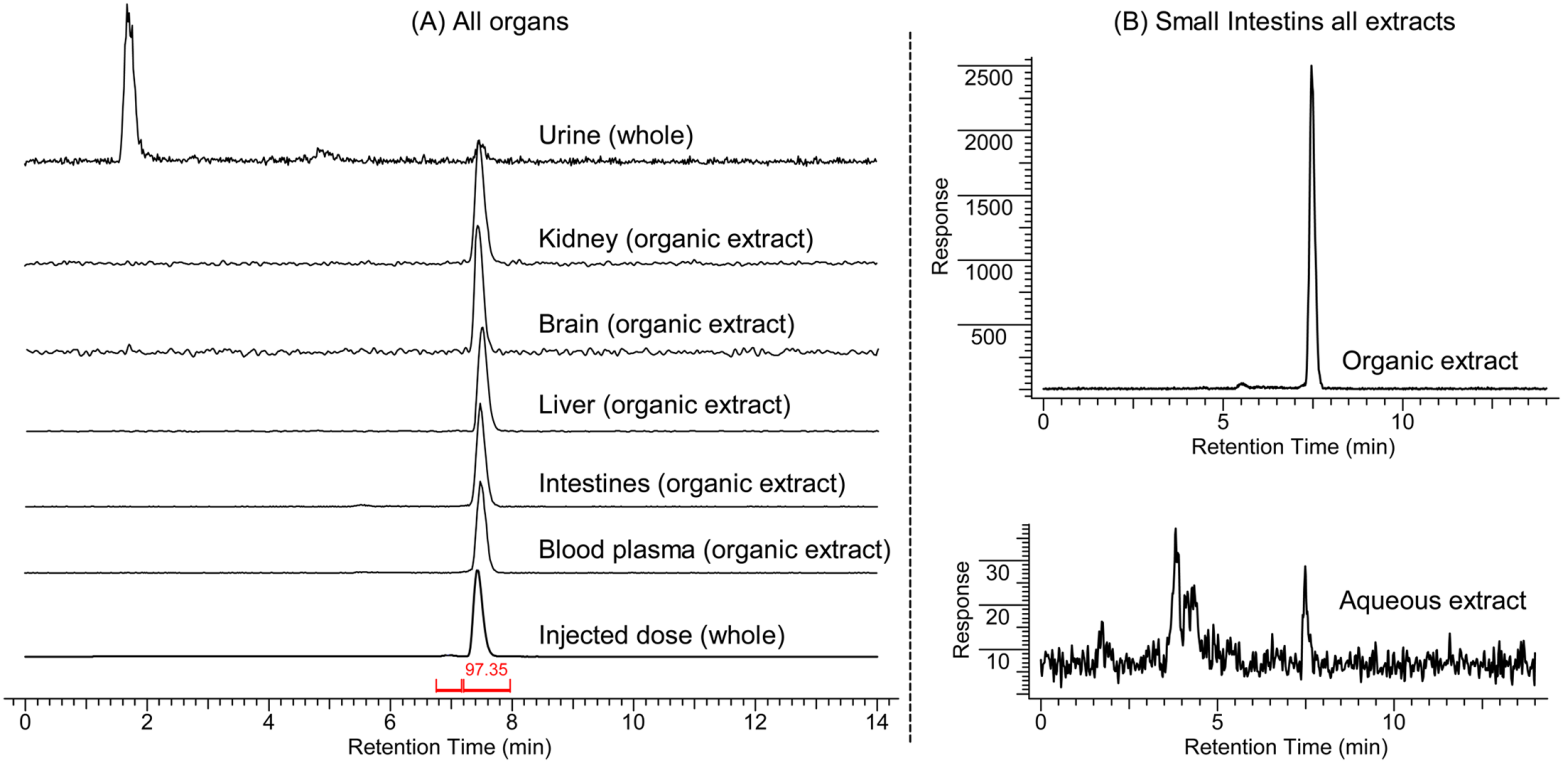
(**A**) Analytical HPLC traces of organic extracts of major organs 1h after administration of 50 uCi of 18F-1. (**B**) Analytical HPLC traces of all extracts of small intestines represented on the same scale.

*In vivo* metabolic stability of the probe was investigated. 1h after bolus injection of ^18^F-**1**, urine, kidney, brain, liver, small intestines and blood plasma were collected from 3 healthy female mice and the radioactivity was extracted and analyzed by HPLC. Experimental protocols are detailed in the supporting information (S5 Protocol). HPLC traces show over 95% intact probe and no discernable metabolites from the radio-HPLC analysis of the organic extracts (Fig 6A). The results of radio-TLC analysis of the same fractions agree with the HPLC traces (see SI). Metabolized radiolabelled molecules were observed in the urine where polar metabolites are excreted, and in the aqueous extracts of the major organs. However, these degradation products accounted for less than 1% of the total activity in all major organs. This is illustrated in (Fig 6B), which presents the chromatograms of the aqueous and organic extracts of small intestines as an example. While the organic fraction is composed of >98% of the parent compound, the aqueous fraction composition includes only approx. 20% of parent in the mixture. However, the amount of radioactivity in the aqueous solution is approximately 1% of the radioactivity in the organic phase, indicating that the metabolites comprise only an insignificant fraction of the total radioactivity.

**Fig 7:**
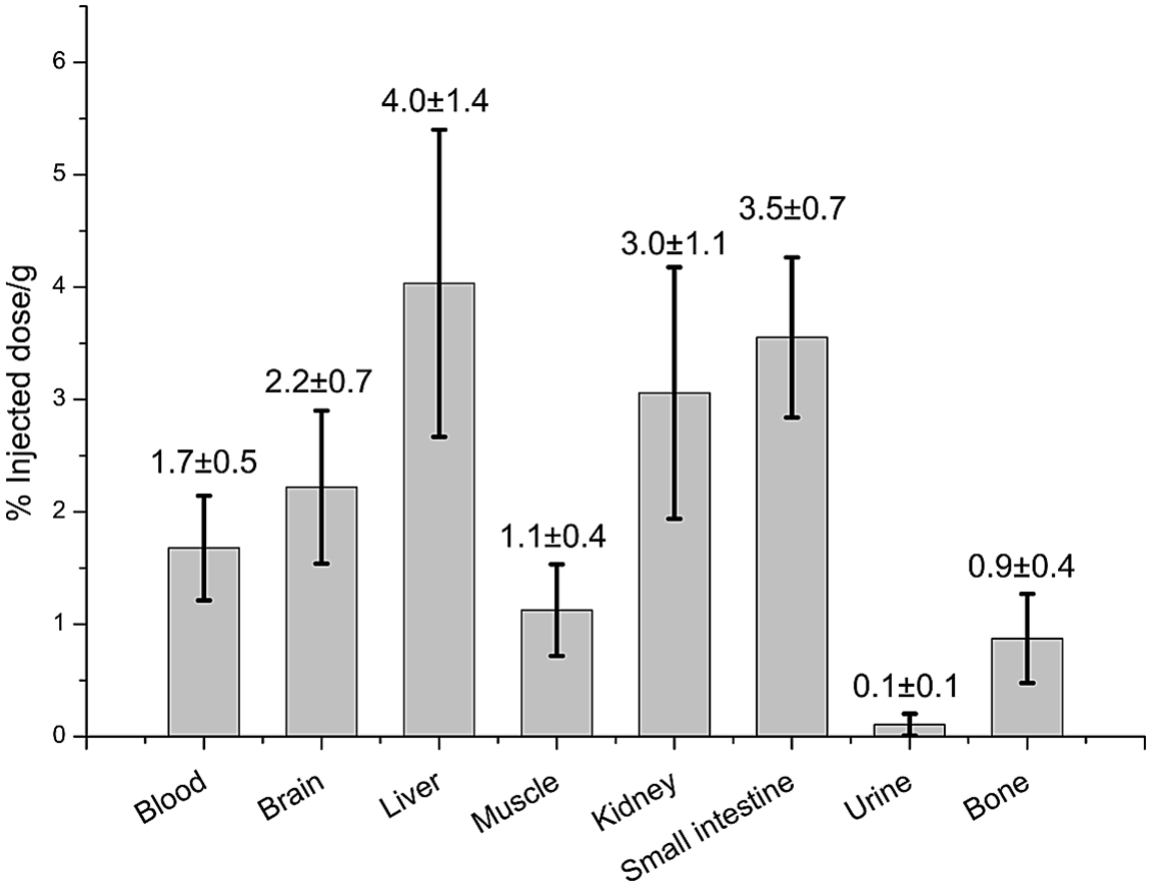
Ex-vivo biodistribution of ^18^F-1. Healthy mice (n = 5) 1 hour after bolus injection.

Ex-vivo biodistribution of the tracer was also studied following a 1 h uptake after administration of the probe. The same organs were collected and radioactivity accumulation was measured and normalized by weight and injected dose. Detailed protocols can be found in supporting information (S4 Protocol). The data presented in Fig 7 show that liver, kidney, and intestines accumulated the most radioactivity. Blood, muscle and brain each accumulated only 1% ID/g. The lowest amount of radioactivity was found in urine and bones.

Static and dynamic PET imaging studies were performed following bolus injection of ^18^F-**1** in healthy wild-type mice. The time series PET images are presented in the supporting information (S4 Protocol) and the region of interest (ROI) analysis from the 60 minutes post injection image is in agreement with the ex-vivo biodistribution data.

## Discussion

### Radioelectrochemical synthesis of COX-2 inhibitor 1

**Fig 8:**
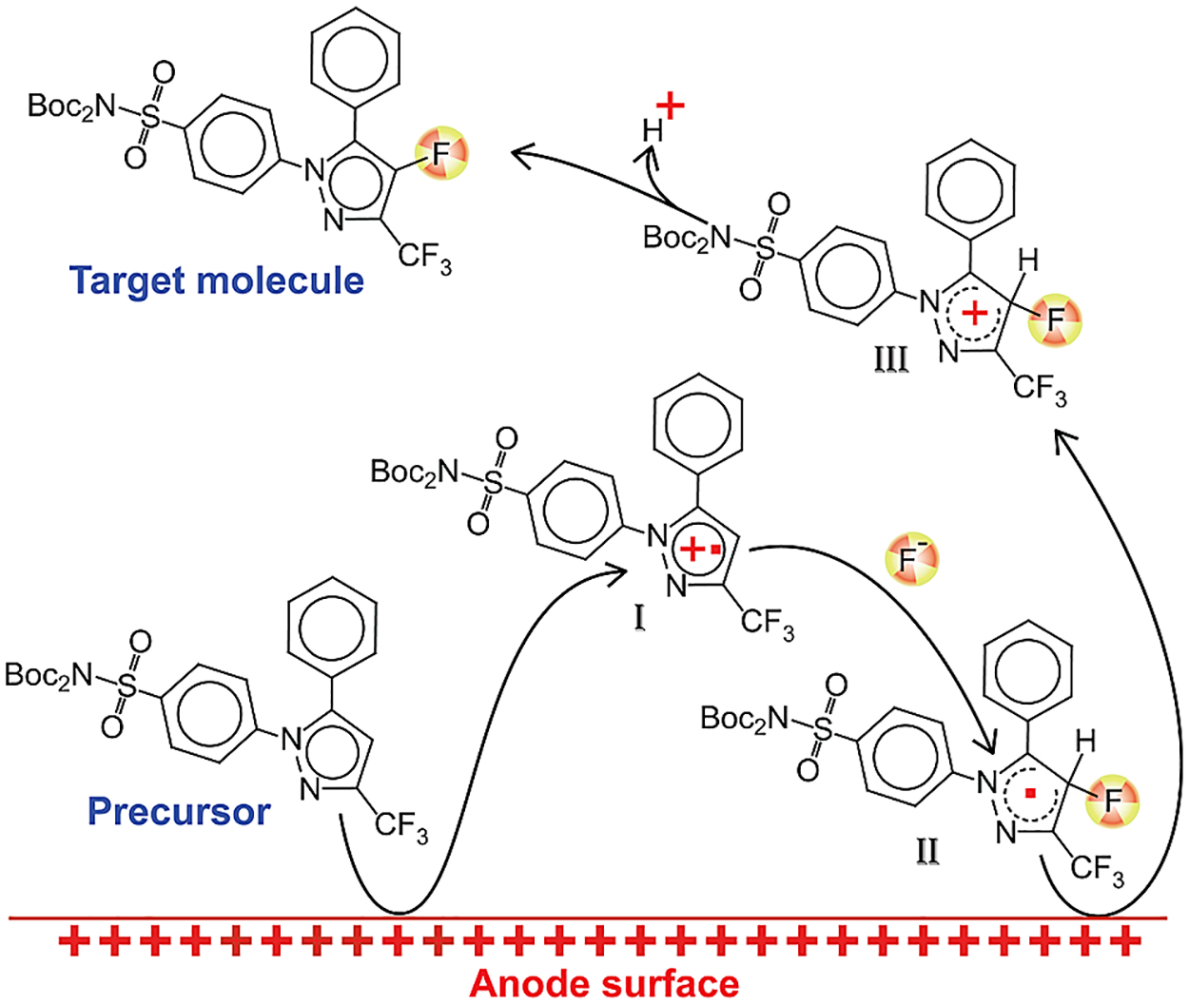
Mechanism of product formation.

The mechanism of the electrochemical fluorination of aromatic compounds has been extensively studied over the past 40 years.[34] The current consensus is that the reaction follows the ECEC sequence presented in Fig 8 and that the mechanism involves four distinct steps: electrochemical oxidation leading to radical cation I in Fig 8, followed by nucleophilic attack of fluoride (II in Fig 8), then another oxidation and the production of the cation III in Fig 8 and subsequent deprotonation and synthesis of the product.

The details of this mechanism dictated the choice of the starting material. Cationic species (**I** in Fig 8) originating at the electrode surface must participate in a bimolecular reaction with fluoride anion in order to form the target product. Monomolecular decomposition of **I** however, can occur immediately after its formation, thus slowing conversion to the target molecule. This reasoning guided selection of protecting groups for the sulfonamide moiety, in that both hydrogen atoms have to be replaced by protecting groups stable toward monomolecular decomposition. Indeed, our attempts to use a precursor having only one Boc protecting group failed to yield any radiofluorinated products (fluoride conversion 0%). Upon substitution of a protecting group for a trityl, 24% fluoride conversion was achieved, but the only fluorinated product was found to be trityl fluoride, most likely due to the high stability of trityl cation that was in turn formed via the same unimolecular decomposition of the cation-radical intermediate.

Development of an automated electrochemical radiosynthesizer platform was a critical element for successful radiosynthesis of the target compound ^18^F-**1**. This was due to large quantities (up to 1000 mCi) of radioactive precursor used in the synthesis, the need to tightly control the electrolysis parameters, and sensitivity of the reaction to moisture and other impurities. Using the in-house built automated platform, we achieved reliable production of the target molecule. More than 20 runs, needed for the studies discussed below, were performed and resulted in up to 5 mCi of the final purified product (2% DCY).

### *In vitro* studies

Reliable supply of the radiolabeled ^18^F-**1** enabled us to probe the factors affecting the *in vitro* cell uptake of the probe. Studies in macrophages demonstrated that the uptake of the tracer increases proportionately to LPS stimulation and can be blocked by excess Celecoxib. These observations illustrate the specificity of ^**18**^**F-1** as a potential imaging agent.

**Fig 9:**
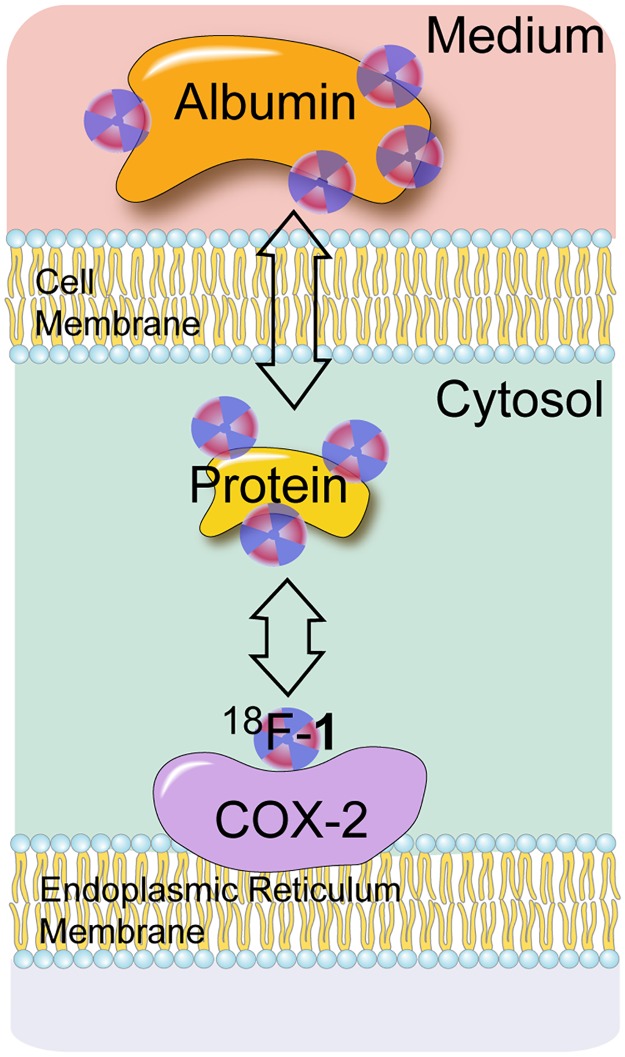
Schematic for association of 18F-1 with COX-2 in cell culture.

Multiple processes can drive accumulation of the probe in the cells. Depending on the cell type, the COX-2 enzyme is localized in the nuclear envelope and/or endoplasmic reticulum.[35],[36] The enzyme itself is a homodimer tethered to the membrane surface via the intermembrane domain.[37] Therefore, before associating with the target protein, ^18^F-**1** has to diffuse through two membranes and cross two cell compartments filled with protein solution. Considering that >95% protein binding of celecoxib in plasma,[38] it is reasonable to expect the tracer, ^**18**^**F-1**, to be bound to one of the cytosolic proteins. Moreover, it has been recently suggested that COX-2 inhibitors have to first enter through the membrane binding domain, before passing through an opening, made exclusively of hydrophobic residues, before entering the active site of the enzyme.[39] Therefore, the diffusion process from the medium to the COX-2 molecule involves many intermediate steps (Fig 9). While available data do not provide any information on the relative rates of these steps, it is clear that dissociation of ^18^F-**1** from the COX-2 molecule is the slowest process. The fact that LPS treatment modulates the uptake of the tracer (Fig 5) is likely due to the increased expression of COX-2 in response to LPS treatment.[40] Close correlation of COX-2 expression measured with western blot and the radioactivity uptake indicates that the uptake saturation is likely due to saturation of COX-2 enzyme expression with increasing LPS, and not because of loss of ^18^F-**1** binding affinity to COX-2. Reduction of uptake in presence of excess celecoxib also points to the fact that association of the probe with the catalytic site of COX-2 enzyme is the driving force in tracer uptake.

### *In vivo* metabolism and PET imaging

Study of the metabolic fate of the compound following *in vivo* administration in mice confirmed that the aromatic position of the fluorine atom confers high *in vivo* stability. This is an improvement over other attempts at creating COX-2 imaging agents. Previous use of the known celecoxib analogs as PET imaging tracers was crippled by low metabolic stability. Up to 83% of radioactivity was found to be metabolized 1 hour after injection in baboons [18]. Our data demonstrates that ^18^F-**1** is free of this drawback.

HPLC analyses of the radioactivity accumulated in all major organs estimated the amount of metabolites 1h post injection to be on the scale of 1% of the amount of the radioactivity present. The great majority of the radioisotope was still present in the form of unchanged parent compound ^18^F-**1**. TLC trace, a method much more sensitive to the presence of polar metabolites, also found no polar metabolites in all major organs.

**Fig 10:**
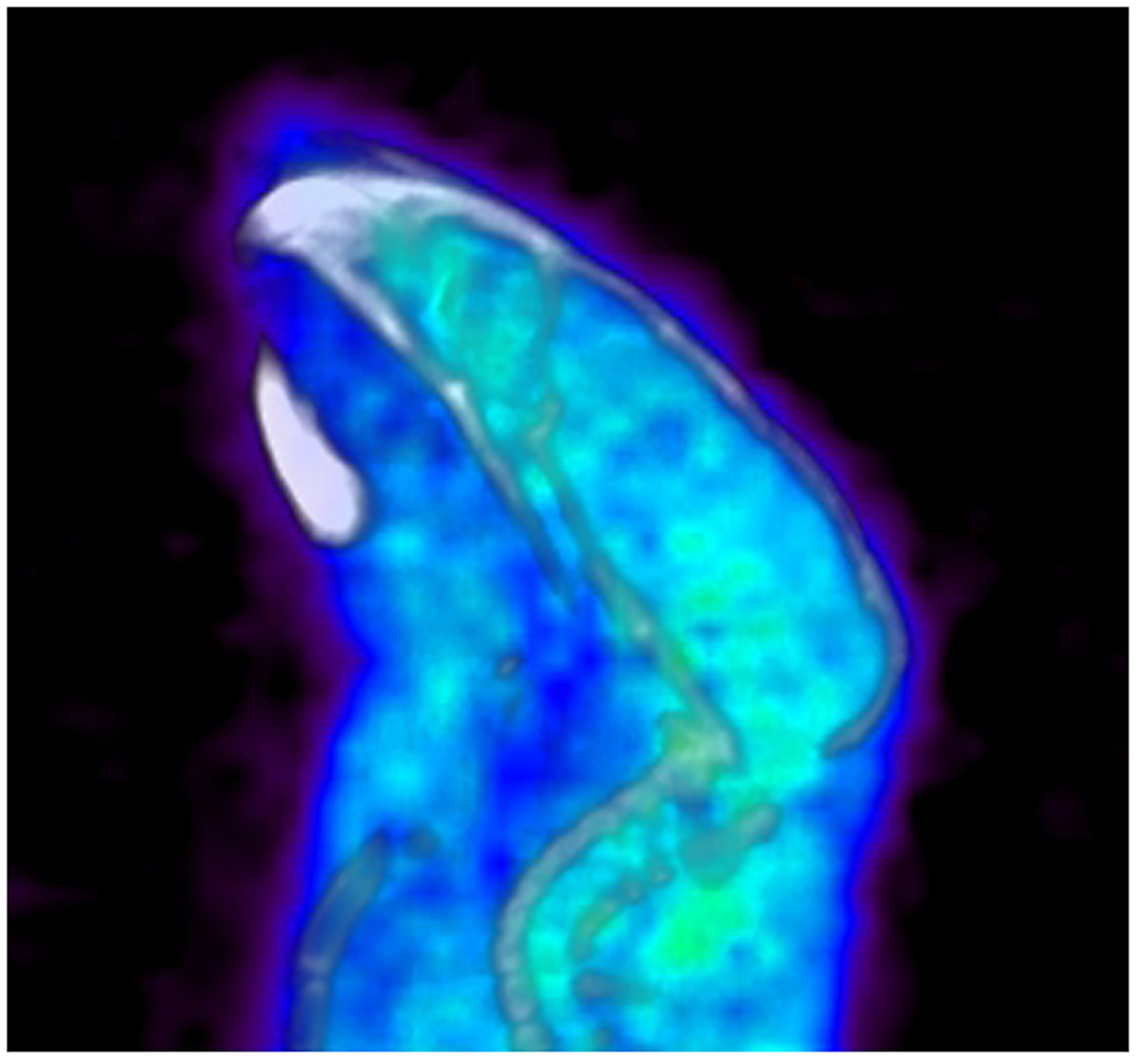
PET/CT image of the mouse head. 90 min after administration of ^18^F-**1**.

Analysis of PET images also supports the notion of high metabolic stability. Fig 10 presents a PET/CT image of the head of a healthy mouse inoculated with ^18^F-**1** 90min prior to the scan. Noticeably, there is no significant increase in ^18^F-**1** retention in the skull or vertebras, where bone uptake if present, would be easily visualized. Due to the small size of the structure, quantitative analysis of the image is not possible, but a characteristic pattern of fluoride uptake in the bone structures,[18] resulting from de-fluorination of the tracer, is not apparent on this image.

Accumulation patterns observed in ex-vivo organ counting agrees with the notion of high metabolic stability as well. Bone uptake, the main indicator of de-fluorination, was at the same level as muscle uptake and below that of blood and brain. Furthermore, there was no free ^18^F-fluoride present in the ex-vivo TLC analysis of the blood.

It is important to note that the metabolic behavior of the tracer is very different from that of celecoxib.[41] While celecoxib is almost completely metabolized through oxidation of the methyl group, compound **1** remains virtually unchanged due to the lack of this substituent. This observation highlights an advantage of ^18^F-**1**, as compared to drugs such as celecoxib, for imaging applications.

**Fig 11:**
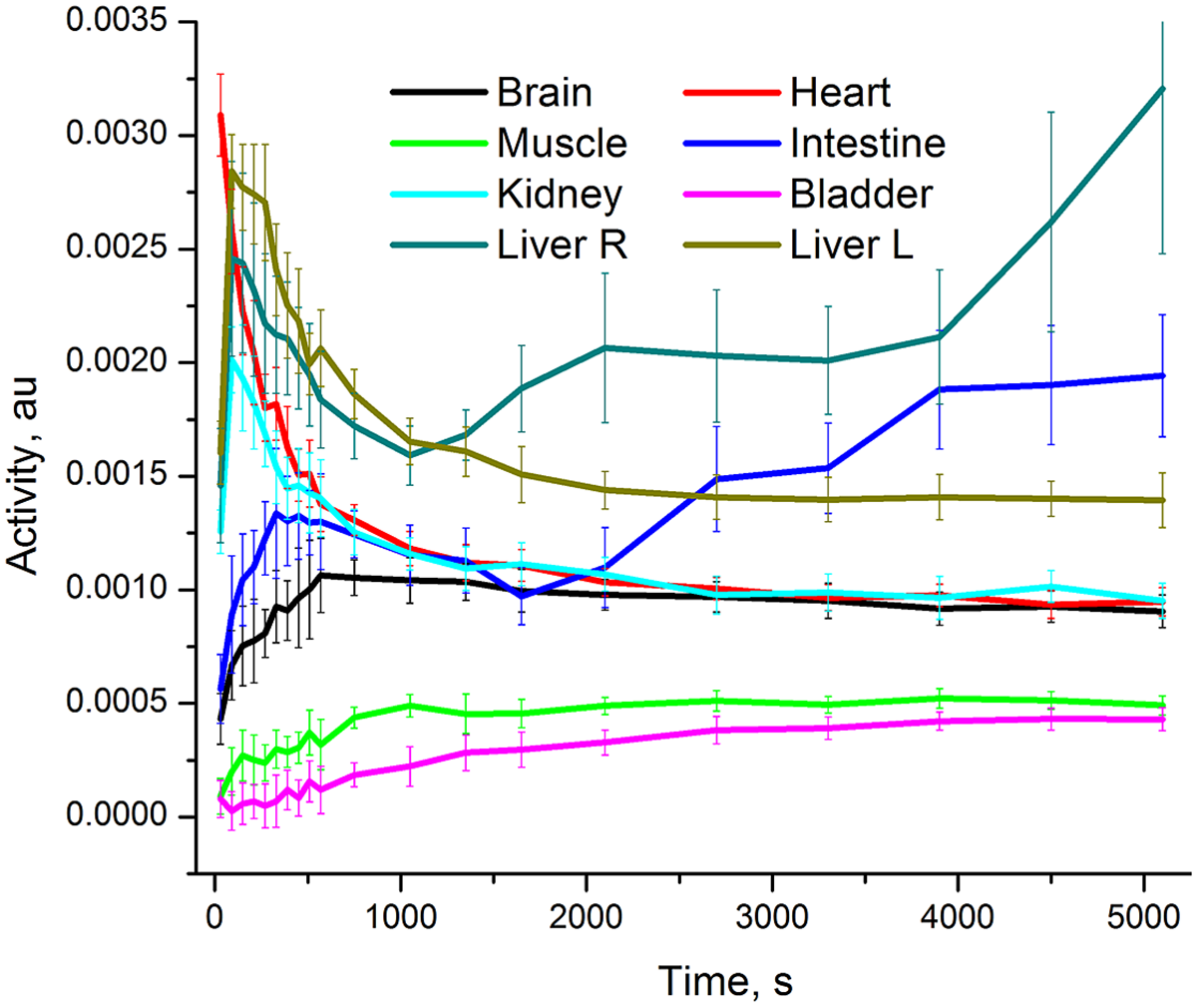
Time-activity profile of major organs. Healthy mouse after bolus iv administration of ^18^F-**1**.

Dynamic PET/CT scans of healthy mice demonstrated favorable pharmacokinetics of ^18^F-**1** for imaging studies with background subsiding within 1 hour and no noticeable defluorination. Fig 11 shows the relative time-activity profiles of all major organs. Within 1 hour after injection concentration of the tracer in all major organs, with the exception of the small intestine and the right side of the liver discussed below, stabilized at a level not exceeding twice that of muscle non-specific uptake. This indicates that there is potential of reaching a good image contrast within 1 hour after injection. Imaging data are in line with ex-vivo biodistribution (Fig 7) with kidney being a notable exception. While the dynamic scan indicates little difference in uptake in the kidney, blood and brain, ex-vivo counting indicates approximately 50% more accumulation in kidney as compared to the brain and blood. The apparent discrepancy between imaging and ex vivo counting data may be due to accumulation of activity in the thin renal cortex excluded from the conservatively defined ROI.[42] Uptake of ^18^F-1 in the kidney may be attributed to known high levels of COX-2 expression in this organ.[43]

Gallbladder is likely the main excretion organ for the tracer. Increase in activity with time in the intestine and low activity in the bladder rule out urinary excretion. Unexpectedly, the left and right sides of the liver exhibit a 2 fold difference of the radioactivity uptake after 90 min. Furthermore, activity in one side of the liver increased with time while the other side of the liver demonstrated a washout rate similar to that of other organs. Additionally, static images reveal boluses of radioactivity distributed along the intestines, likely due to accumulation in feces. A similar accumulation pattern has been reported previously in human subjects for other 18F labeled tracers, where larger image sizes allowed for positive identification of gallbladder as the main excretion organ. [44]

Of particular interest is the time-activity curve of the brain ROI. Following the initial ramp up, brain radioactivity concertation decreases and is then stabilized at a level similar to other organs and twice the uptake of muscle. This is likely due to initial diffusion across the blood brain barrier and subsequent washout of the tracer from brain tissue. This indicates the potential applicability of this molecule for brain imaging of COX-2 expression, which is of particular interest for imaging of neurodegenerative diseases.[45]

## Conclusion

Early detection and imaging of COX-2 overexpression would be an important clinical tool for monitoring disease progression, therapy intervention, evaluation of therapeutic treatment efficacy, and identification of patients who can be selected for COX-2 inhibitor or NSAID treatment. A COX-2 PET probe could potentially replace invasive biopsy and should reduce sampling errors due to target heterogeneity, as well as instability of COX-2 protein and mRNA, and should help determine the pharmacokinetics and *in vivo* binding characteristics of new COX-2 inhibitors.

The probe structure of ^18^F-**1** is based on some of the lessons learned over the last two decades of research in developing a viable COX-2 PET tracer. To address the challenges of *in vivo* stability, we have placed ^18^F on a stable heteroaromatic ring in a structure that maintains high COX-2 affinity and specificity, using a synthesis that is practical and made possible through the electrochemical platform. Electrochemical synthesis, performed on the automated platform developed in our lab, is readily adaptable for use by imaging researchers without requiring expertise in electrochemistry. Pharmacokinetic studies with ^18^F-**1** in healthy mice revealed no bone retention or defluorination within 2 hours of injection, significant blood clearance, crossing of the BBB and no significant metabolites in major organs, making the probe ideally suited for imaging studies.

The current decay corrected radiochemical yield of 2%, although enough for preclinical studies and production of a single patient dose, needs improvement. More importantly, the use of Et_4_NF*4HF has limited the specific activity to 3 Ci/mmol, severely limiting the application of the probe as a successful imaging agent. We anticipate that the use of microfluidics and electrochemical flow cells for this surface activated reaction will increase the yield and specific activity. To address the specific activity problem, we are investigating the use of alternative quaternary ammonium or phosphonium salts of non-nucleophilic anions, polar and solvating solvents and reducing cation reactivity through cold temperature electrolysis and stabilized cation pools.[46]

## Supporting information

S1 Protocol Synthetic procedures for precursor and standards. (DOCX)

S2 Protocol Radioelectrochemistry protocol and experimental set up. (DOCX)

S3 Protocol In vitro cell association studies protocol. (DOCX)

S4 Protocol Ex vivo biodistribution studies and imaging protocol and data. (PDF)

S5 Protocol Metabolism study protocol. (DOCX)

S1 Spectra Spectroscopic and chromatographic data for all stable and radioactive compounds. (PDF)
