# Supplementary material for: Radiochemistry on electrodes: Synthesis of an 18F-labelled and in vivo stable COX-2 inhibitor
Source: PLoS One. 2017 May 2;12(5):e0176606. doi: 10.1371/journal.pone.0176606 (PMC5413030; doi:10.1371/journal.pone.0176606)
Supplement: S1 Protocol — (DOCX) [file pone.0176606.s001.docx]

# Synthesis of precursors and standards.

**Pyrazole S1**. 12 N HCl (0.73 mL, 8.76 mmol, 0.125 mol/L) was added to a mixture of 1,3-dione (10.80 g, 50.0 mmol) and phenylhydrazine hydrochloride (11.15 g, 50.0 mmol) in N,N-dimethylacetamide (50 mL) at r.t. under argon. The reaction was stirred for 24 h at r.t. before extraction in the mixture of petroleum ether (50 mL), ethyl acetate (150 mL), and water (250 mL). The organic phase was washed with NaHCO_3_ sat. aq. (30 mL) and water (3 × 250 mL) before it was dried over Na_2_SO_4_, filtered, and concentrated under vacuum to give an oil, which was again dissolved in acetone (50 mL) and xylene (200 mL), and then evaporated in vacuum in order to remove the remaining N,N-dimethylacetamide. Recrystallization in heptane/ethyl acetate afforded the product as white or off-white needle crystals (14.95 g, 40.7 mmol, yield 82%).

**Pyrazole S2**. Sulfonamide and DMAP were dissolved in 100 mL of DCM in a 250 mL flask. Boc_2_O was measured out in a separate glass beaker and then added to the reaction flask neat. Boc_2_O container was additionally washed with 20 mL of DCM and the solution was added to the reaction mixture. Vigorous gas formation started immediately, but no noticeable temperature change was observed. Reaction mixture was refluxed for 1 hour, then dry silica (approx. 20 ml) was added and the mixture was evaporated to dryness. The resulting silica powder was applied to a medium size silica column (~30 x 3 cm). The column was eluted sequentially with pure Hexane, 5% EtOAc in hexane and 10 % EtOAc in Hexane. The product was collected in a 10% EtOAc fraction. The solvent was evaporated on rotary evaporator at 60^o^C in a water bath. To avoid foam formation while drying, the residue was kept under low vacuum for 2 hours before final drying under high vacuum pump. 2.05 g (36%) of product in the form of a clear glassy solid was obtained after overnight drying. The product slowly crystallized over several weeks at room temperature.

Pyrazole **S3** was synthesized according to the procedure described for **S1**.
