# Supplementary material for: Radiochemistry on electrodes: Synthesis of an 18F-labelled and in vivo stable COX-2 inhibitor
Source: PLoS One. 2017 May 2;12(5):e0176606. doi: 10.1371/journal.pone.0176606 (PMC5413030; doi:10.1371/journal.pone.0176606)
Supplement: S2 Protocol — (DOCX) [file pone.0176606.s002.docx]

# Electrochemical radiofluorination

Details of the design and performance of the automated electrochemical radiosynthesis platform are described in a separate manuscript under preparation. The radiochemical yields reported in this manuscript are based on the radioactivity measurements from the purified, injectable tracers compared to starting activities from the cyclotron and take into account losses in the lines, syringes and synthesis platform. Figure 1 illustrates the fluidic diagram of the automated electrochemical radiosynthesizer.


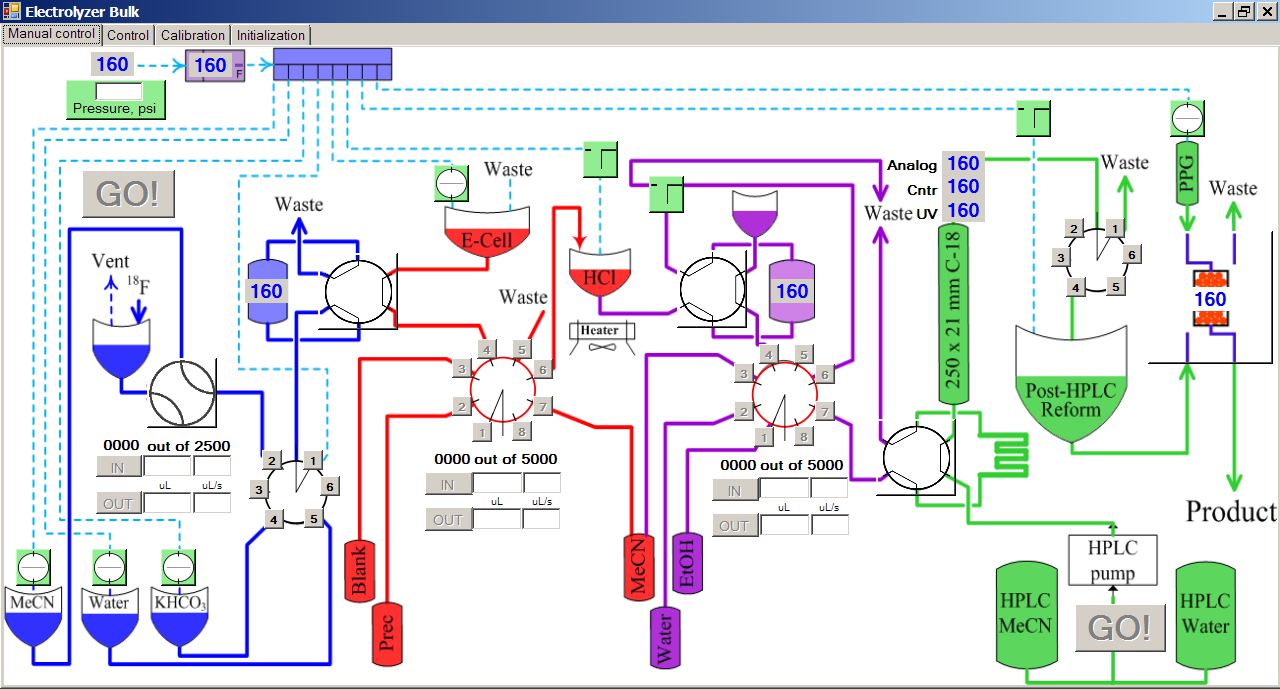


**Figure 1.** Fluidic diagram of the in-house build radioelectrochemical synthesizer.

**Figure 2.** HPLC calibration for compounds **S2**, **S3.** Data for compound 1 from the final injectable formulation is presented.

Specific activity was determined by using compounds **S2** and **S3** as standard. Isolation of compound **1** in quantitates sufficient for analytical determination of concentration proved to be difficult and the HPLC system was calibrated using compounds S2 and S3. Since the calibration curves shown in **Figure *2*** , for these two compounds do not significantly differ, we assumed that calibration for the compound **1** will be very similar as well.
