# Supplementary material for: Radiochemistry on electrodes: Synthesis of an 18F-labelled and in vivo stable COX-2 inhibitor
Source: PLoS One. 2017 May 2;12(5):e0176606. doi: 10.1371/journal.pone.0176606 (PMC5413030; doi:10.1371/journal.pone.0176606)
Supplement: S3 Protocol — (DOCX) [file pone.0176606.s003.docx]

# In vitro uptake experiments

Radioactivity of all samples was measured using “Wizard 3" automatic gamma counter (Perkin Elmer)

## Radioactivity dose escalation studies

Due to the carrier added nature of the synthesis, excessive activity during the *in vitro* uptake protocol would lead to blocking by ^19^F-**1** and too little activity would introduce noise in the data. The following protocol was used to find the optimum radioactivity concentration:

1. 0.2*10^6^ RAW 264.7 cells were plated the day before the experiment in 2.5 ml of media in each well of the respective 6-well plates (**Figure *3***)
2. LPS (2000 ng/ml) in was added to the wells according to scheme on (**Figure *3***) and the plates were incubated overnight at 37^o^C in 5% CO2 atmosphere.
3. On the day of experiments four serial dilutions of **^18^F-1** were prepared in media starting from 0.6 mC/ml solution in 50% polypropylene glycole:

**Tracer 1**: 0.6 uCi/ml

**Tracer 2** 0.06 uCi/ml

**Tracer 3** 0.006 uCi/ml

**Tracer 4** 0.0006 uCi/ml

1. Add 500ul of the respective **Tracer** to all 6 wells in the respective plate
2. Incubate plates at 37^o^C, 5%CO_2_ for 1 h
3. Collect 1 ml of media
4. Wash out residue of the tracer by washing the cells twice with pure conditioned media
5. Add 1 ml of Trypsin solution and incubate for 5 min
6. Collect cell suspensions in counting tube
7. Add additional 500ul Trypsin to well to remove remaining cells, combine the wash with the respective suspension Step 13.
8. Count all samples and a 20 uL of the **Tracer** in the gamma counter at 5 min/sample.

Plate 4

Plate 1

**LPS = 2000 ^ng^**/**_mL_**

**LPS = 0 ^ng^**/**_mL_**

Plate 2

Plate 3

**LPS = 2000 ^ng^**/**_mL_**

**LPS = 0 ^ng^**/**_mL_**

**LPS = 2000 ^ng^**/**_mL_**

**LPS = 0 ^ng^**/**_mL_**

**LPS = 2000 ^ng^**/**_mL_**

**LPS = 0 ^ng^**/**_mL_**

**^18^F-1: 0.1** **^uCi^**/**_mL_**

**^18^F-1: 0.01** **^uCi^**/**_mL_**

**^18^F-1: 0.001** **^uCi^**/**_mL_**

**^18^F-1: 0.0001** **^uCi^**/**_mL_**

**Figure 3. Schematic of the dose escalation experiment**

**Figure 4. Radioactivity concentration dependent cell uptake of ^18^F-1 in LPS stimulated macrophages**

## LPS dose escalating study

The following protocol was followed to elucidate the influence of LPS concentration on the uptake of **^18^F-1**:

1. RAW 264.7 (mouse leukaemic monocyte macrophage cell line) cells were plated with 0.2x106 cells/well in 6-well plate, and treated with different concentration of LPS (0, 50, 100, 250, 500,1000ng/ml, LPS from E. coli serotype 055:B5; Sigma, L-6529) for 16 hrs. (**Figure 5**)
2. On the day of experiments three solutions were prepared using conditioned media at 37^o^C and 5%CO_2_:
   1. **Tracer** 0.06 uCi/mL **^18^F-1** in conditioned media for addition to plates 1-5
   2. **Vehicle** DMSO 100 uL/ml in conditioned media for addition to plate 4
   3. **Celecoxib** mix 4.76 ml of conditioned media and add 0.24ml celecoxib (stock 4 mg/ml) for final conc = 32ug/ml celecoxib during uptake in plate 5
3. Aspirate 500 uL of media from all wells in Plates 4 and 5
4. Add 500 uL of **Celexocib** to plate 5
5. Add 500 uL of **Vehicle** to plate 4
6. Incubate plates at 37^o^C, 5%CO_2_ for 30 min.
7. Add 500ul concentrated tracer media (0.06 uCi/mL **^18^F-1**) to 2.5mL in each well, note start and end times (final dose = 0.01uCi/mL)
8. Incubate samples at 37^o^C, 5%CO_2_ for 1 h
9. Remove media
10. Wash out residue of the tracer by washing the cells twice with pure conditioned media
11. Add 1 ml of Trypsin solution and incubate for 5 min
12. Collect cell suspensions in counting tube
13. Add additional 500ul Trypsin to well to remove remaining cells – add to respective sample in Step 9.
14. Count all samples and a 1mL of the master mix in one run for proper decay correction.

**Figure 5. LPS treatment schematic.**

Plate 5

**LPS: 1000 ^ng^**/**_mL_**

**Celecoxib: 32 ^ug^**/**_ml_**

**LPS: 0 ^ng^**/**_mL_**

**Celecoxib: 32 ^ug^**/**_ml_**

Plate 4

**LPS: 1000 ^ng^**/**_mL_**

**DMSO: 20 ^ul^**/**_well_**

**LPS: 0 ^ng^**/**_mL_**

**DMSO: 20 ^ul^**/**_well_**

Plate 1

**LPS = 50 ^ng^**/**_mL_**

**LPS = 0 ^ng^**/**_mL_**

Plate 2

**LPS = 250 ^ng^**/**_mL_**

**LPS = 100 ^ng^**/**_mL_**

Plate 3

**LPS = 1000 ^ng^**/**_mL_**

**LPS = 500 ^ng^**/**_mL_**

## Western Blot determination of COX-2 expression

RAW 264.7 (mouse leukaemic monocyte macrophage cell line) cells were plated with 0.2x106 cells/well in 6-well plate, and treated with different concentration of LPS (0, 50, 100, 250, 500,1000ng/ml, LPS from E. coli serotype 055:B5; Sigma, L-6529) for 16 hrs. Cells treated with each concentration (4 wells) were split in half: two wells used for Western analysis, two wells used for COX-2 PET probe assay. For western analysis, RAW cells lysate was homogenized in RIPA buffer containing a proteinase inhibitor cocktail (Santa Cruz, sc-24948) and incubated at 4oC for 20 minutes on a rocking platform. Cell debris was removed by centrifugation and protein content was determined by Bradford assay. Proteins (25ug) were separated on 10% SDS-PAGE gels and transferred onto nitrocellulose membranes. The membranes were blocked with 5% milk protein in TBS/0.1% Tween-20, probed with primary antibodies in the same buffer over night at 4oC, then incubated with anti-rabbit HRP-conjugated secondary antibody (Santa Cruz, sc-2004, 1:5000) for one hour at room temperature. Proteins were visualized on autoradiographic film using ECL reagent (Pierce). The following antibodies were used for Western blotting: COX-2 (Santa Cruz, sc-1747-R), 1:1000; GAPDH (Santa Cruz, sc-25778), 1:1000
