# Supplementary material for: Radiochemistry on electrodes: Synthesis of an 18F-labelled and in vivo stable COX-2 inhibitor
Source: PLoS One. 2017 May 2;12(5):e0176606. doi: 10.1371/journal.pone.0176606 (PMC5413030; doi:10.1371/journal.pone.0176606)
Supplement: S4 Protocol — (PDF) [file pone.0176606.s004.pdf]

1. Ex-vivo counting protocol

^{18}F-**1** biodistribution in wild-type C57BL6 mice was confirmed ex vivo. Mice were sacrificed after 1 h uptake time using 5% isoflurane with cervical dislocation. Tissues were collected, weighed and radioactivity counted on a Wizard 3" automatic gamma counter (Perkin Elmer).

2. Imaging protocol and data

Animal studies were approved by the UCLA Animal Research Committee and were carried out according to the guidelines of the Division of Laboratory Animal Medicine at UCLA. C57BL6 mice underwent ^{18}F-**1** PET/CT imaging studies were performed using the GENISYS 8 PET/CT (G8, Sofie Biosciences), and Inveon microPET (Siemens Medical Solutions). Mice were anesthetized under 2% isoflurane, placed in a heated G8 imaging chamber and catheterized by tail vein. The chamber was placed in the PET section of the G8 or Inveon. PET images were acquired from 0-1 hr (dynamic) or at 1 hr (static) after bolus injection of ^{18}F-**1** (approximately 2.8 MBq for G8 and 4.6 MBq for Inveon). G8 images were reconstructed using maximum-likelihood expectation maximization as recommended by the vendor and Inveon images were reconstructed with a zoom factor of 2.1 using 3D-OSEM with 2 iterations followed by MAP with 18 iterations (beta=0.1). All images were corrected for photon attenuation, detector normalization and radioisotope decay, and converted to units of percent injected dose per gram (%ID/g). CT images are acquired in a continuous-rotation mode with 720 projections at 55 msec per projection, and reconstructed using a Feldkamp algorithm.

Images were analyzed using AMIDE version 1.0.5 e.g. with 2mm diameter 3D elliptical regions-of-interest (ROI). ROI were determined based on the anatomical CT information.

Representative time series of coronal slice is presented below.

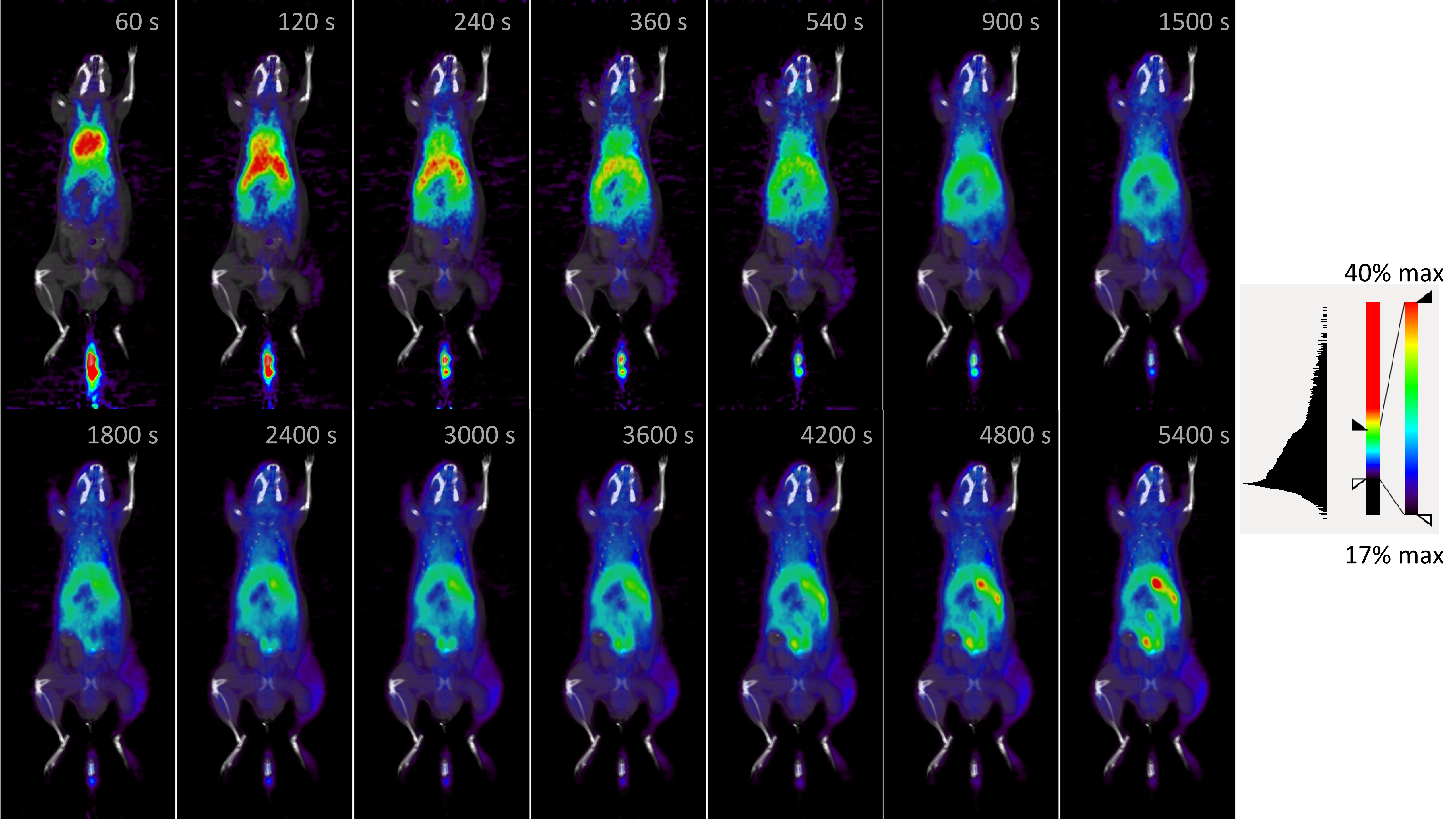
60 s
120 s
240 s
360 s
540 s
900 s
1500 s
1800 s
2400 s
3000 s
3600 s
4200 s
4800 s
5400 s
40% max
17% max
