# Supplementary material for: Radiochemistry on electrodes: Synthesis of an 18F-labelled and in vivo stable COX-2 inhibitor
Source: PLoS One. 2017 May 2;12(5):e0176606. doi: 10.1371/journal.pone.0176606 (PMC5413030; doi:10.1371/journal.pone.0176606)
Supplement: S5 Protocol — (DOCX) [file pone.0176606.s005.docx]

# Metabolism studies protocol

Animal studies were approved by the UCLA Animal Research Committee and were carried out according to the guidelines of the Division of Laboratory Animal Medicine at UCLA. C57BL6 female mice received bolus injection of ^18^F-**1** (approx. 4.0 MBq) in the tail vein while awake. The animals were sacrificed by CO_2_ suffocation after 1 hour uptake time. The organs were collected, weighed and mixed with approx. equal weight of 1% SDS solution. The organs were homogenized in SDS using a mechanical organ homogenizer. The homogenate was subjected to 1 freeze-thaw cycle at -80C. 1 ml of the homogenate was mixed with 1 ml of 1:1 mixture of CHCl_3_ and MeOH. The resulting suspension was vortexed for 2 min and then centrifuged at 10000 rpm for 10 min. Top (aqueous) and bottom (organic) layers were collected using a hand-held pipette and analyzed using HPLC and TLC.

TLC analysis was performed using 100 mm aluminum backed silica gel strips and 95% MeCN -5% water eluent. HPLC analysis was performed using 2 150 mm columns (Agilent ZORBAX Eclipse XDB C18) coupled together for increased resolution. Mobile phase was MeCN/Water 50 to 75% over 15 min.
