# Supplementary material for: Radiochemistry on electrodes: Synthesis of an 18F-labelled and in vivo stable COX-2 inhibitor
Source: PLoS One. 2017 May 2;12(5):e0176606. doi: 10.1371/journal.pone.0176606 (PMC5413030; doi:10.1371/journal.pone.0176606)

4-[5-phenyl-3-(trifluoromethyl)-1*H*-pyrazol-1-yl]benzenesulfonamide: ^{1}H NMR

Acquisition Time (sec)	3.2998	***Comment***	Account No. YYZ002 H2NO2S proton			***Date***	21 Feb 2014 23:06:40
File Name	D:\Google Drive\UCLA\My Data\2014 02 19\NMR\Proton\Proton_fid					***Frequency (MHz)***	400.13
Nucleus	1H	***Number of Transients***	8	***Original Points Count***	26441	***Points Count***	131072
Pulse Sequence	zg30	***Solvent***	DMSO-D6	***Sweep Width (Hz)***	8012.82	***Temperature (degree C)***	26.001

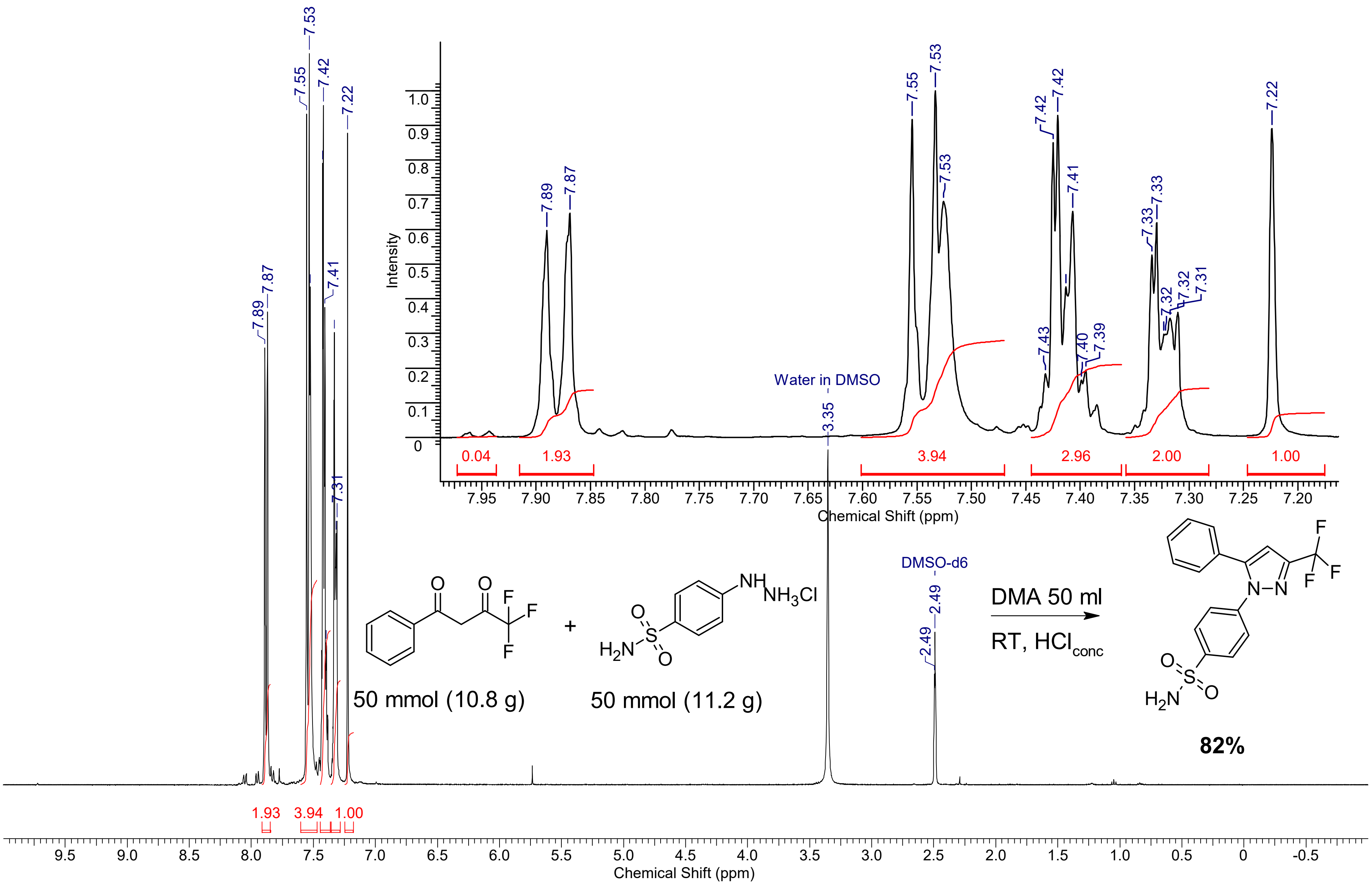

4-[5-phenyl-3-(trifluoromethyl)-1*H*-pyrazol-1-yl]benzenesulfonamide: ^{13}C NMR

Acquisition Time (sec)	1.2976	***Comment***	Account No. YYZ002 H2NO2S carbon			***Date***	21 Feb 2014 23:21:36
File Name	D:\Google Drive\UCLA\My Data\2014 02 19\NMR\Carbon\Carbon_fid					***Frequency (MHz)***	100.62
Nucleus	13C	***Number of Transients***	256	***Original Points Count***	32768	***Points Count***	131072
Pulse Sequence	zgpg30	***Solvent***	DMSO-D6	***Sweep Width (Hz)***	25252.53	***Temperature (degree C)***	26.425

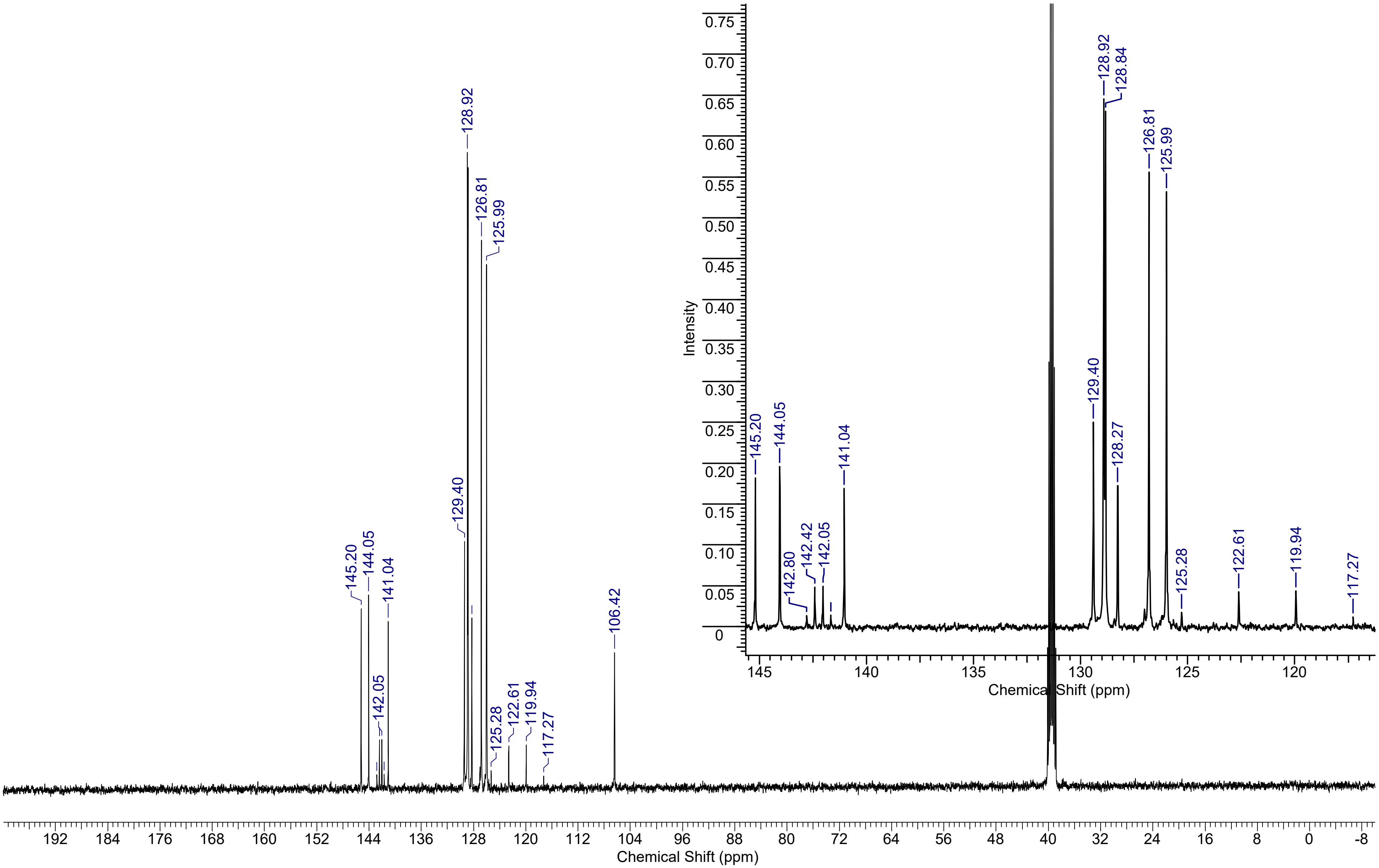

4-[5-phenyl-3-(trifluoromethyl)-1*H*-pyrazol-1-yl]benzenesulfonamide: ESI MS

Count	253	***Data Type***	Centroided Mass Spectrum			***Date Stamp***	09:00:00
File Name	C:\GDRIVE\PUBLISHING\2015 COX-2\COMPOUNDS\PH, H2NO2S-PH, CF3 PYRAZOLE\LCMS\SULPHMSV.AIA\MSSIG01.CDF						
Inlet Model	Electrospray Inlet	***Instrument***	Agilent Technologies / 1946A /			***Ion Mode***	ES+
Operator	EN	***Owner***	EN	***Retention Time***	17.052	***Scan***	1303
Separation Type	Other Chromatography	***Spectrum Title***	Results	***TIC***	190.70	***Total Signal***	705157

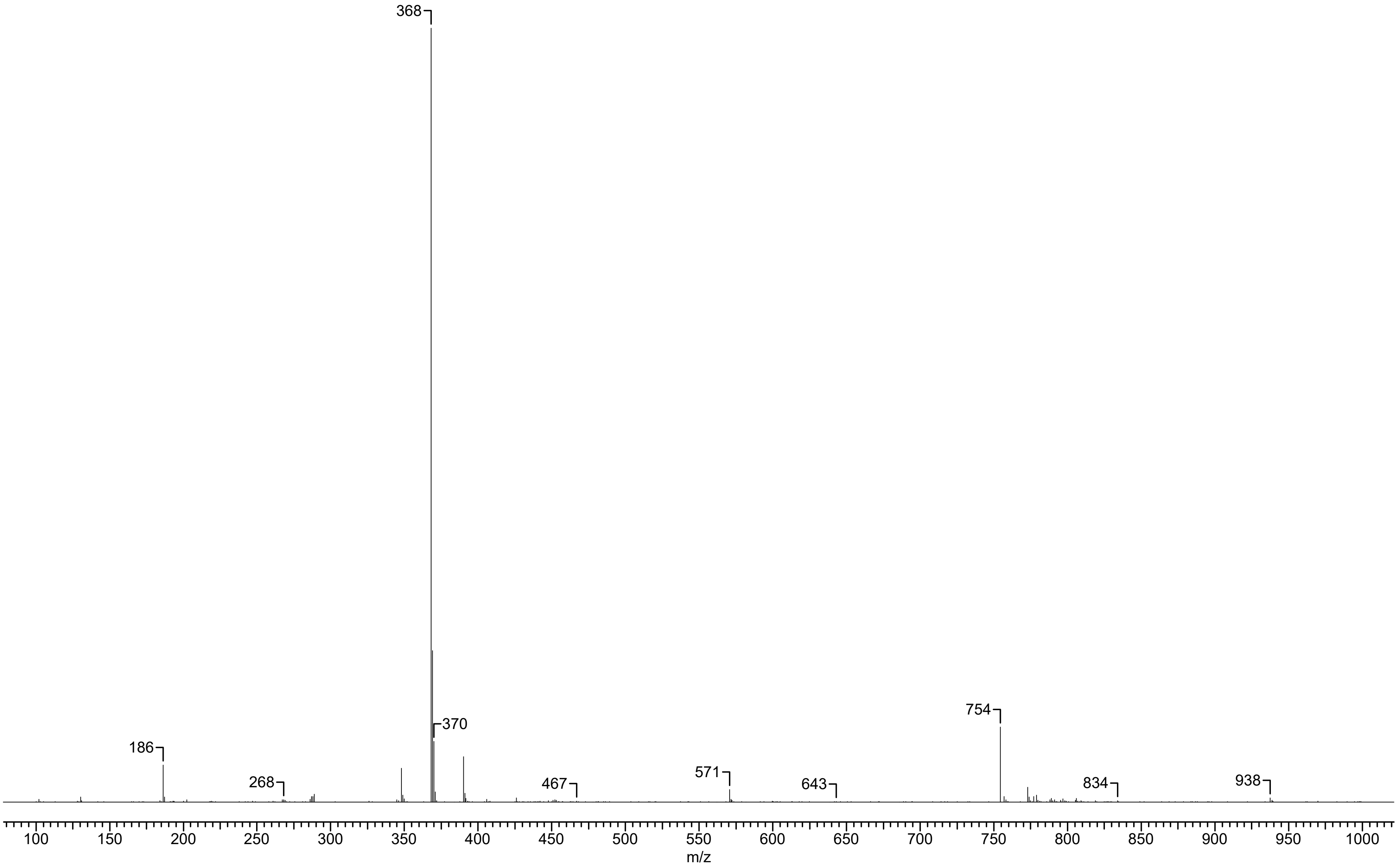

di-*tert*-butyl ({4-[5-phenyl-3-(trifluoromethyl)-1*H*-pyrazol-1-yl]phenyl}sulfonyl)imidodicarbonate: ^{1}H NMR

Acquisition Time (sec)	3.2998	***Comment***	Account No. YYZ002 Boc2NO2SPh, Ph, CF3 proton		
Date	03 May 2014 00:27:44				
File Name	D:\Google Drive\UCLA\My Data\2014 04 15 Ph, Boc2NO2S-Ph, CF3 pyrazole\2014 04 15 Ph, Boc2NO2S-Ph, CF3 pyrazole_590000fid				
Frequency (MHz)	400.13	***Nucleus***	1H	***Number of Transients***	8
Original Points Count	26441	***Points Count***	262144	***Pulse Sequence***	zg30
Solvent	DMSO-D6	***Sweep Width (Hz)***	8012.82	***Temperature (degree C)***	26.535

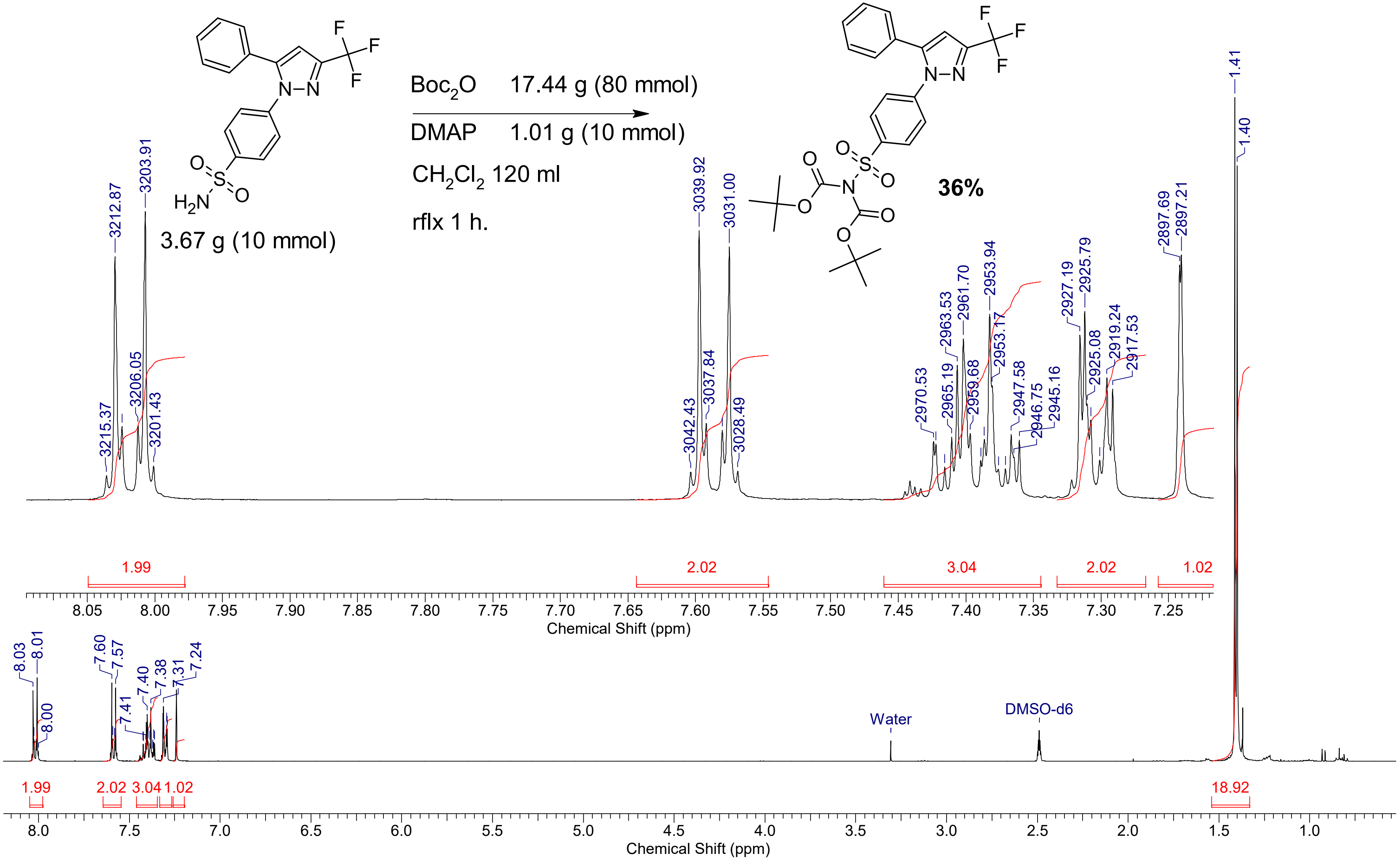

di-*tert*-butyl ({4-[5-phenyl-3-(trifluoromethyl)-1*H*-pyrazol-1-yl]phenyl}sulfonyl)imidodicarbonate: ^{13}C NMR

Acquisition Time (sec)	1.2976	***Comment***	Account No. YYZ002 Boc2NO2SPh, Ph, CF3 carbon			***Date***	03 May 2014 00:44:48
File Name	D:\Google Drive\UCLA\My Data\2014 04 15\2014 04 15_591000fid			***Frequency (MHz)***	100.62	***Nucleus***	13C
Number of Transients	256	***Original Points Count***	32768	***Points Count***	262144	***Pulse Sequence***	zgpg30
Solvent	DMSO-D6	***Sweep Width (Hz)***	25252.53	***Temperature (degree C)***	28.210		

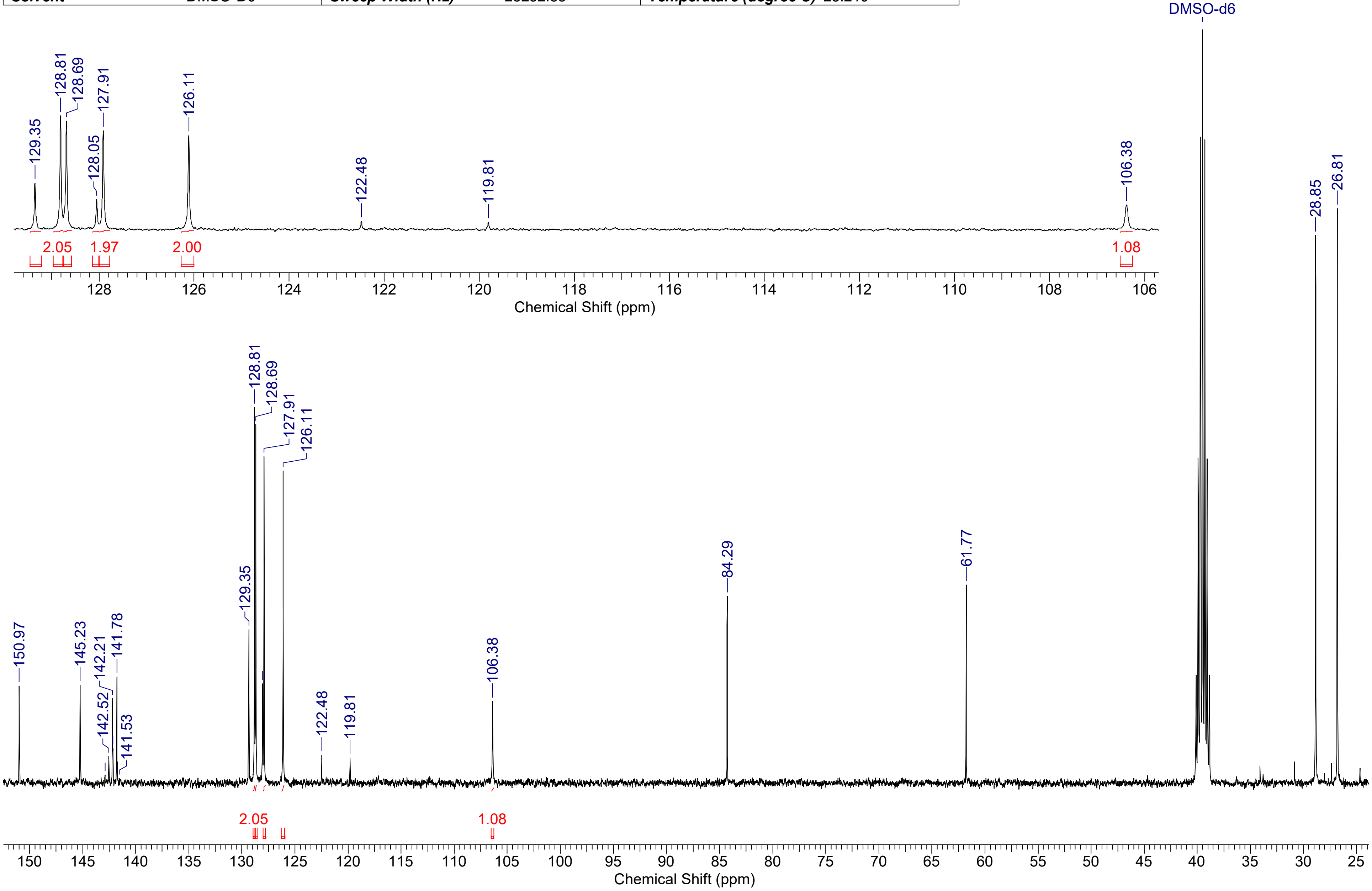

di-*tert*-butyl ({4-[5-phenyl-3-(trifluoromethyl)-1*H*-pyrazol-1-yl]phenyl}sulfonyl)imidodicarbonate: ^{19}F NMR

Acquisition Time (sec)	0.8738	***Comment***	Account No. YYZ002 Boc2NO2SPh, Ph, CF3 fluorine			***Date***	03 May 2014 00:46:56
File Name	D:\Google Drive\UCLA\My Data\2014 04 15\2014 04 15_592000fid			***Frequency (MHz)***	376.50	***Nucleus***	19F
Number of Transients	32	***Original Points Count***	131072	***Points Count***	131072	***Pulse Sequence***	zgfhigqn.2
Solvent	DMSO-D6	***Sweep Width (Hz)***	150000.00	***Temperature (degree C)***	27.744		

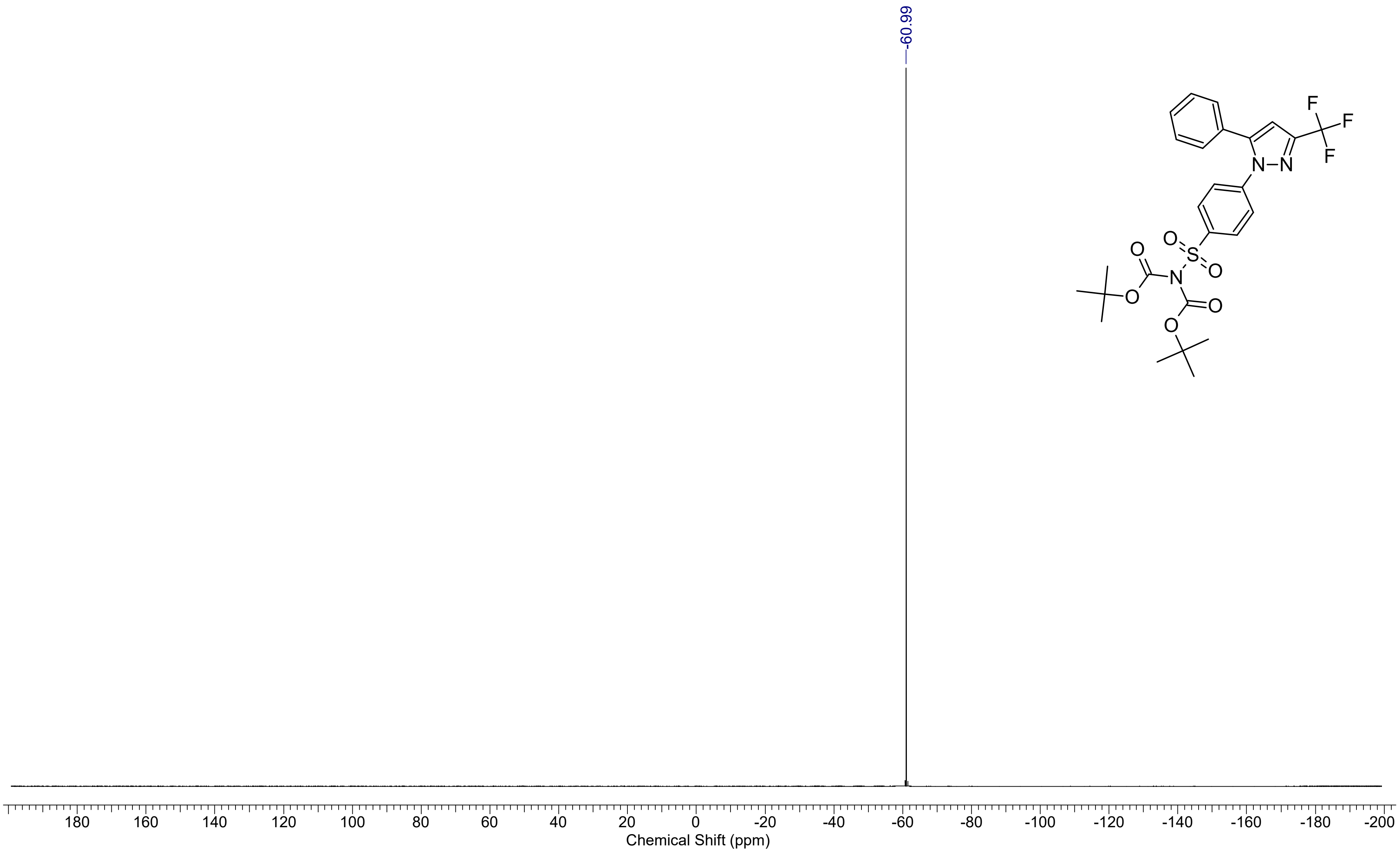

4-[4-fluoro-5-phenyl-3-(trifluoromethyl)-1*H*-pyrazol-1-yl]-benzenesulfonamide (^{19}F-1): ^{1}H-NMR

Acquisition Time (sec)	3.2998	Comment	Account No. YYZ002 F-posicoxib proton			Date	09 Jul 2014 22:43:12
File Name	D:\GDrive\Publishing\2015 COX-2\Compounds\Compound 1\NMR\Proton AV400\Proton AV400_fid					Frequency (MHz)	400.13
Nucleus	1H	Number of Transients	8	Original Points Count	26441	Points Count	65536
Pulse Sequence	zg30	Solvent	DMSO-D6	Sweep Width (Hz)	8012.82	Temperature (degree C)	26.045

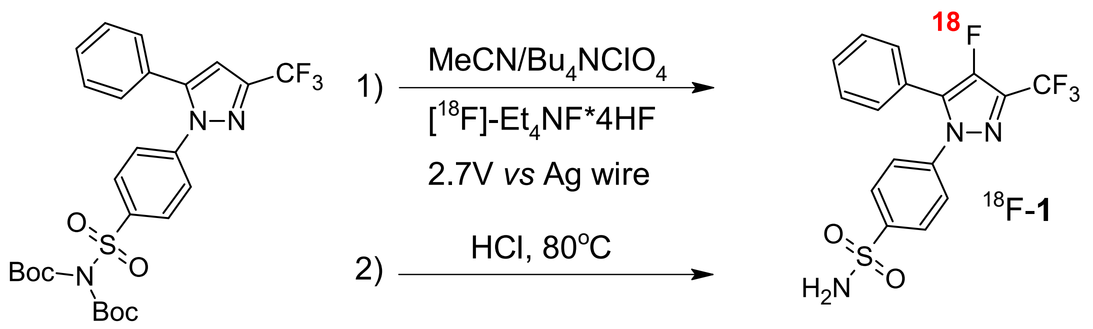

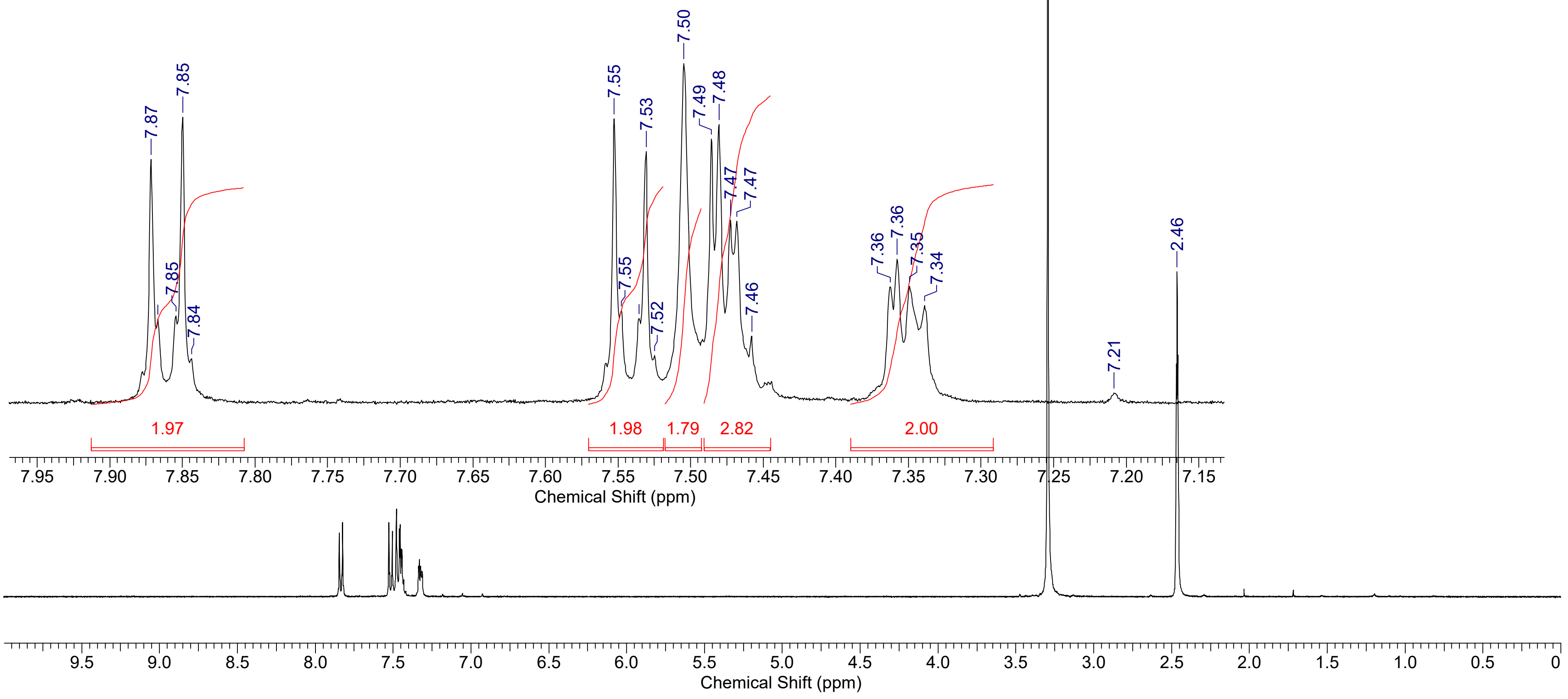

4-[4-fluoro-5-phenyl-3-(trifluoromethyl)-1*H*-pyrazol-1-yl]-benzenesulfonamide (^{19}F-1): ^{19}F NMR

Acquisition Time (sec)	0.8738	***Comment***	Account No. YYZ002 Posicoxib fluorine			***Date***	09 Jul 2014 22:51:44
File Name	D:\GDrive\Publishing\2015 COX-2\Compounds\Compound 1\NMR\Fluorine AV400\Fluorine AV400_fid					***Frequency (MHz)***	376.50
Nucleus	19F	***Number of Transients***	8	***Original Points Count***	131072	***Points Count***	131072
Pulse Sequence	zgflqn30	***Solvent***	DMSO-D6	***Sweep Width (Hz)***	150000.00	***Temperature (degree C)***	26.036

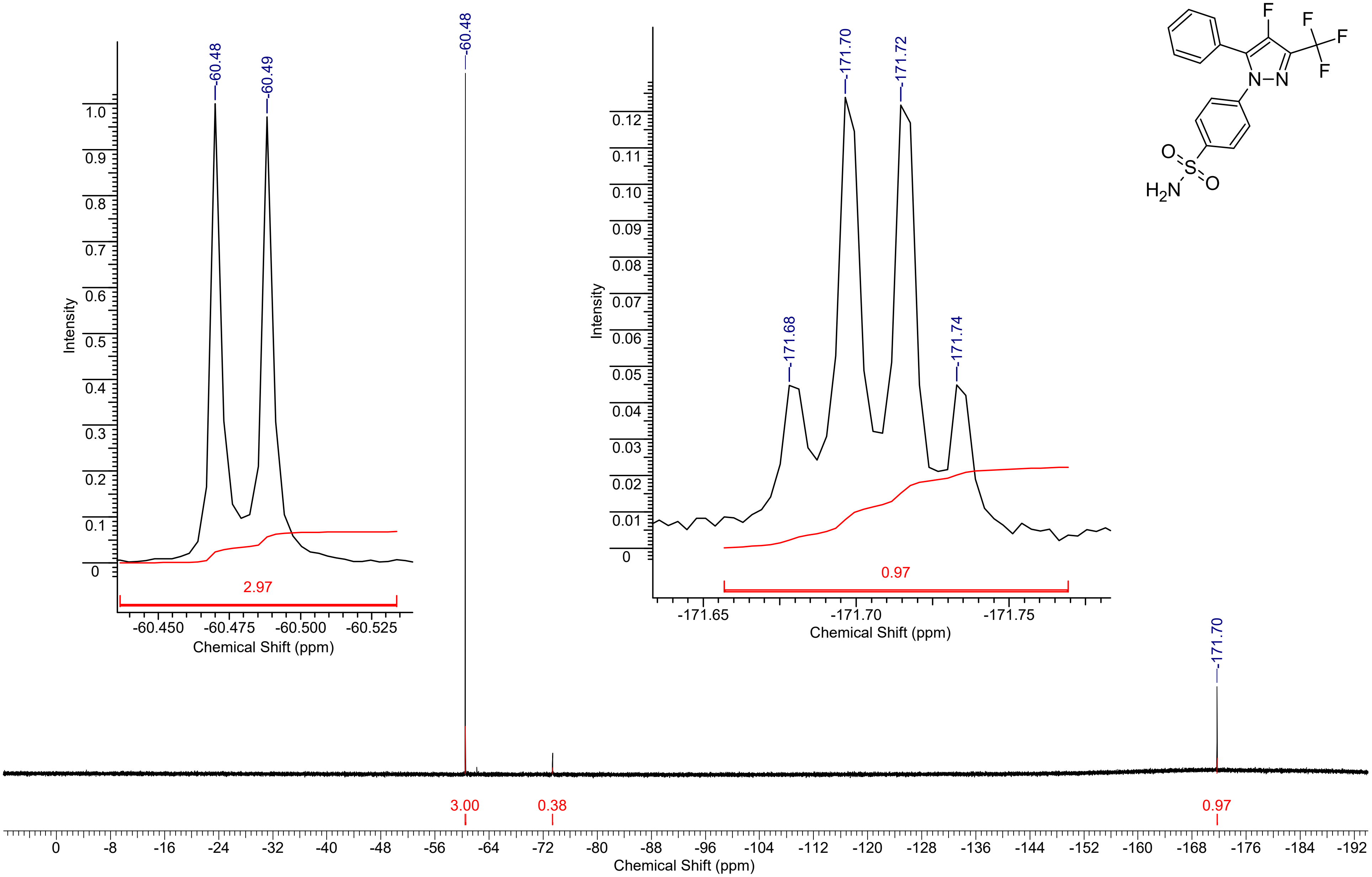

4-[4-fluoro-5-phenyl-3-(trifluoromethyl)-1*H*-pyrazol-1-yl]-benzenesulfonamide (^{19}F-1): ^{13}C NMR

Acquisition Time (sec)	1.0486	***Date***	11 Jul 2014 18:50:40				
File Name	D:\GDrive\Publishing\2015 COX-2\Compounds\Compound 1\NMR\Carbon AV500\Carbon AV500_fid					***Frequency (MHz)***	125.77
Nucleus	13C	***Number of Transients***	840	***Original Points Count***	32768	***Points Count***	32768
Pulse Sequence	zgpg30	***Solvent***	DMSO-D6	***Sweep Width (Hz)***	31250.00	***Temperature (degree C)***	25.001

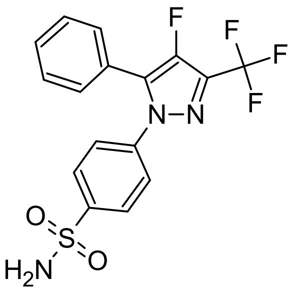

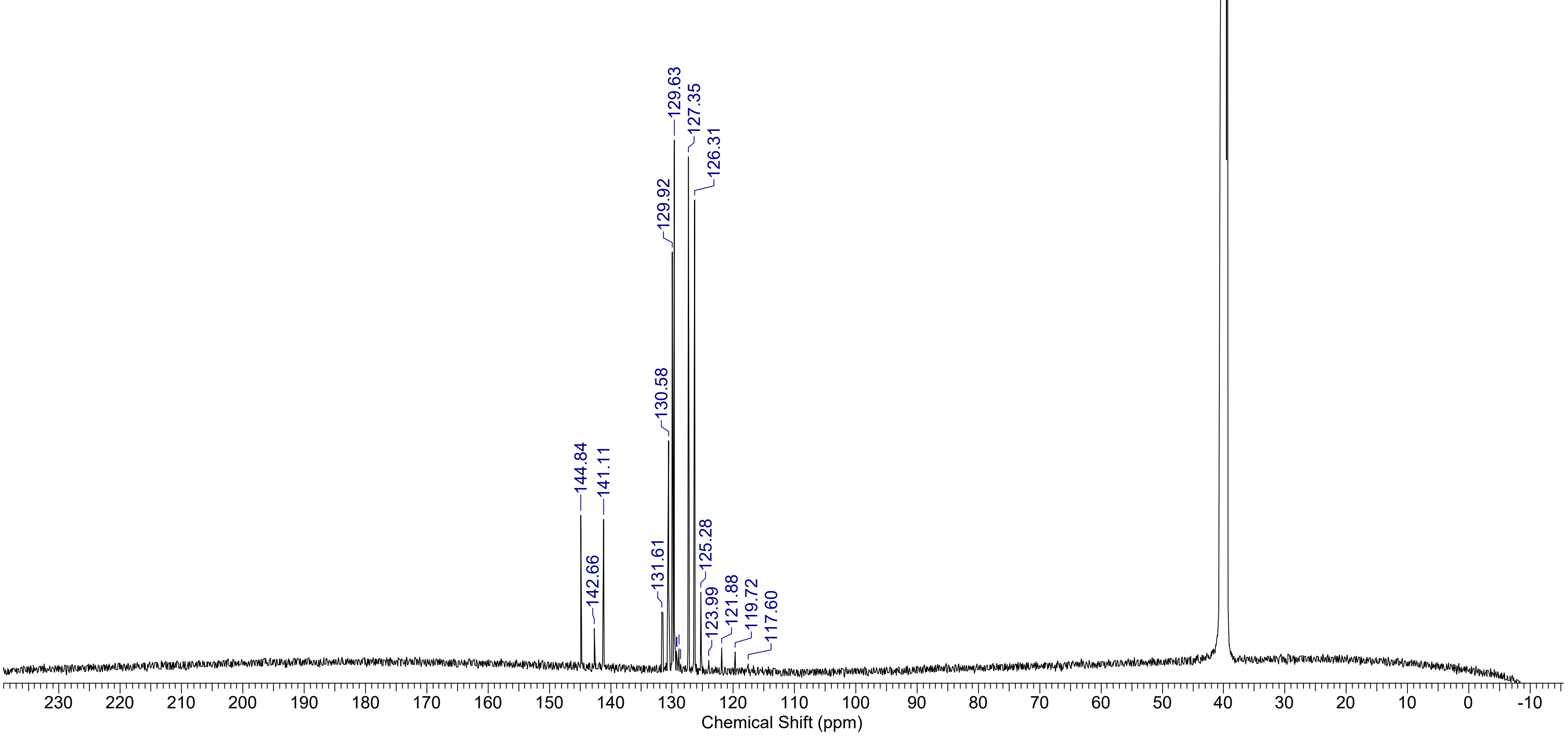

4-[4-fluoro-5-phenyl-3-(trifluoromethyl)-1*H*-pyrazol-1-yl]-benzenesulfonamide (^{19}F-1): ^{13}C NMR

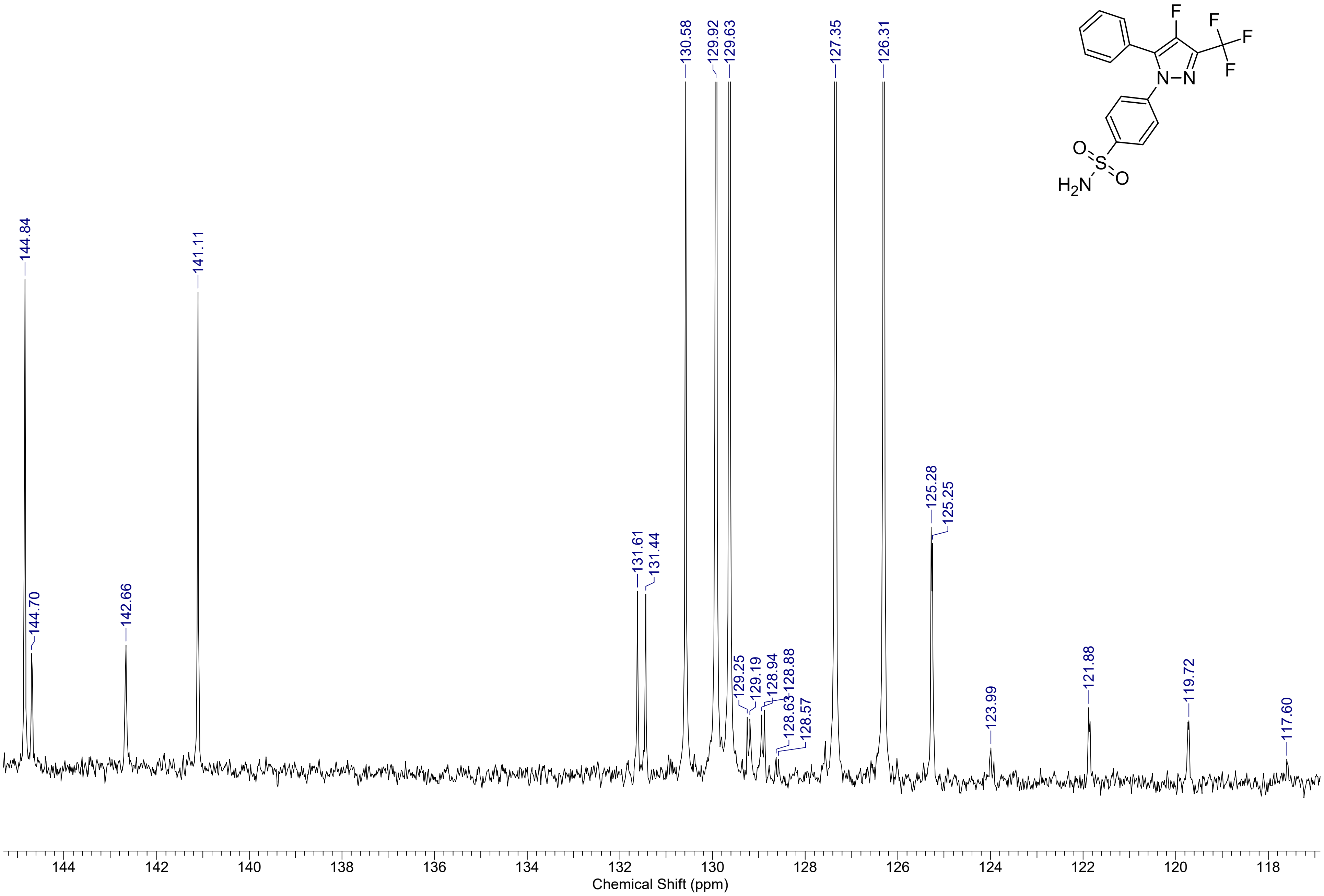

4-[4-fluoro-5-phenyl-3-(trifluoromethyl)-1*H*-pyrazol-1-yl]-benzenesulfonamide (^{19}F-1): ESI MS

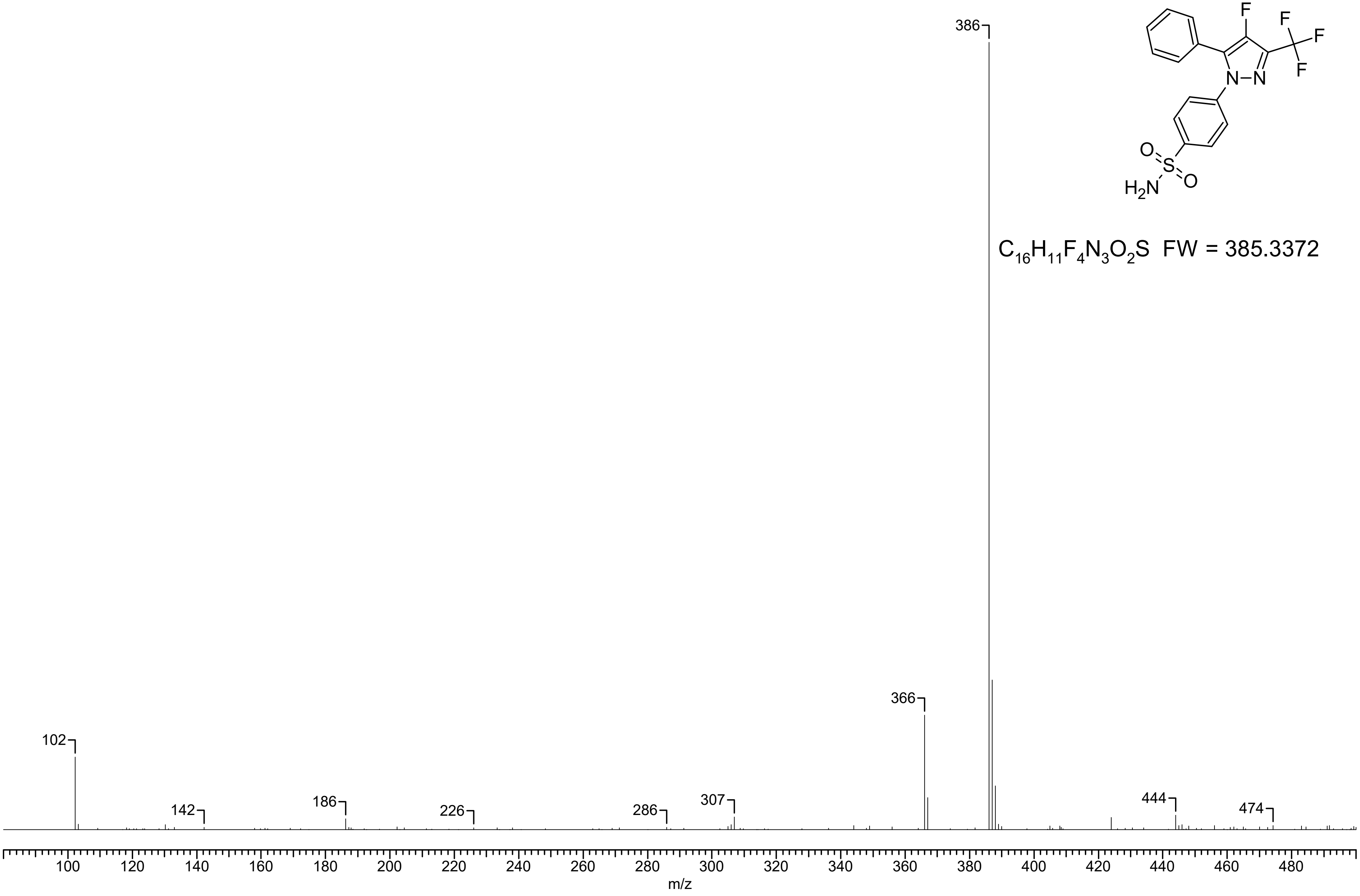

$C_{16}H_{11}F_4N_3O_2S$ FW = 385.3372

4-[4-fluoro-5-phenyl-3-(trifluoromethyl)-1*H*-pyrazol-1-yl]-benzenesulfonamide ([19]F-1): Ananlytical HPLC

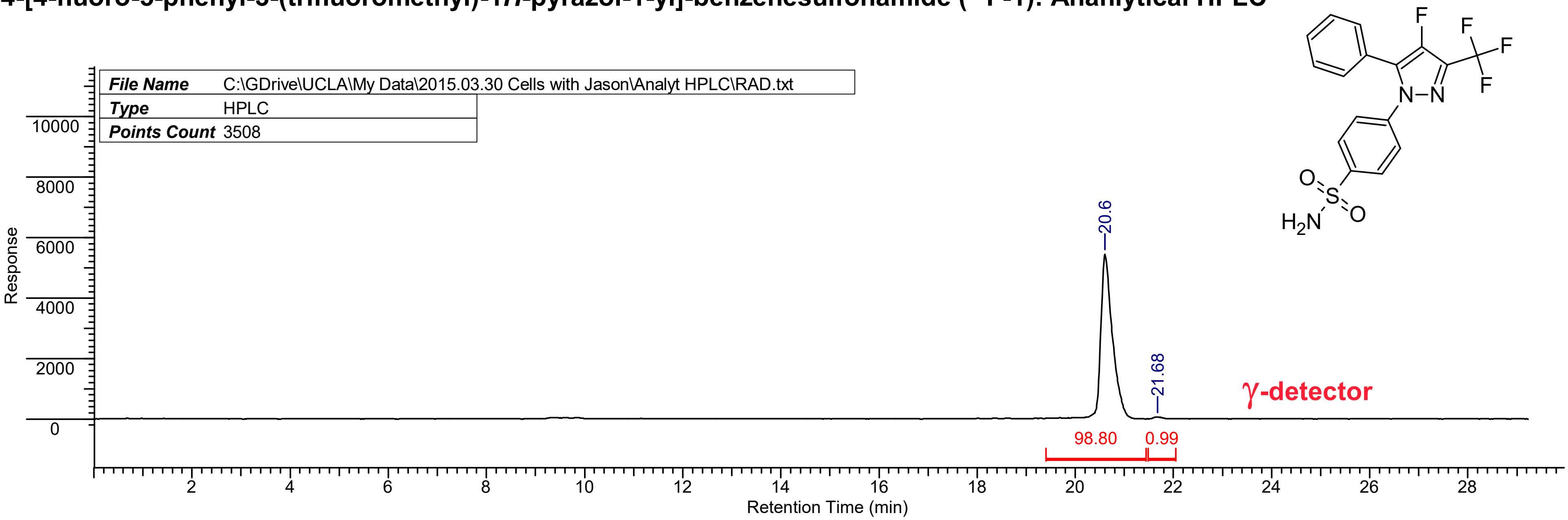

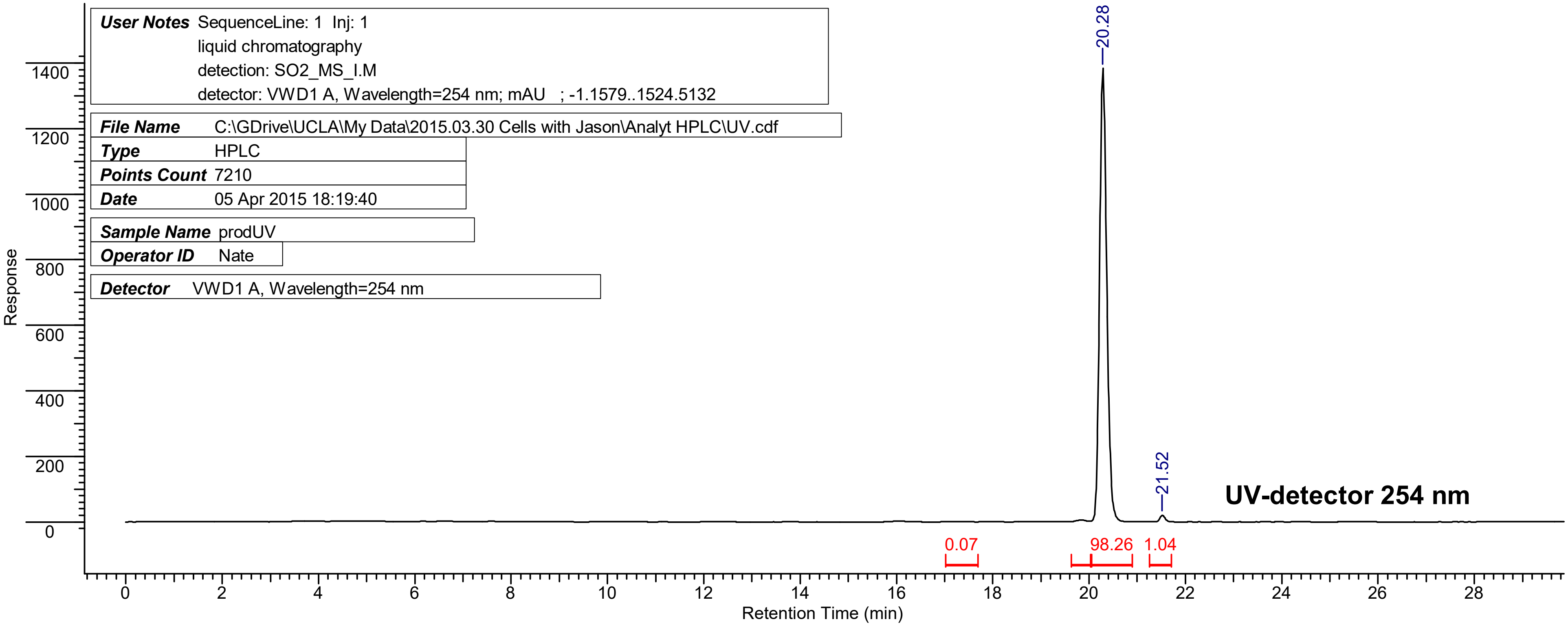

Compound S3: UV-254, Total ion chromatogramms and ESI MS spectrum

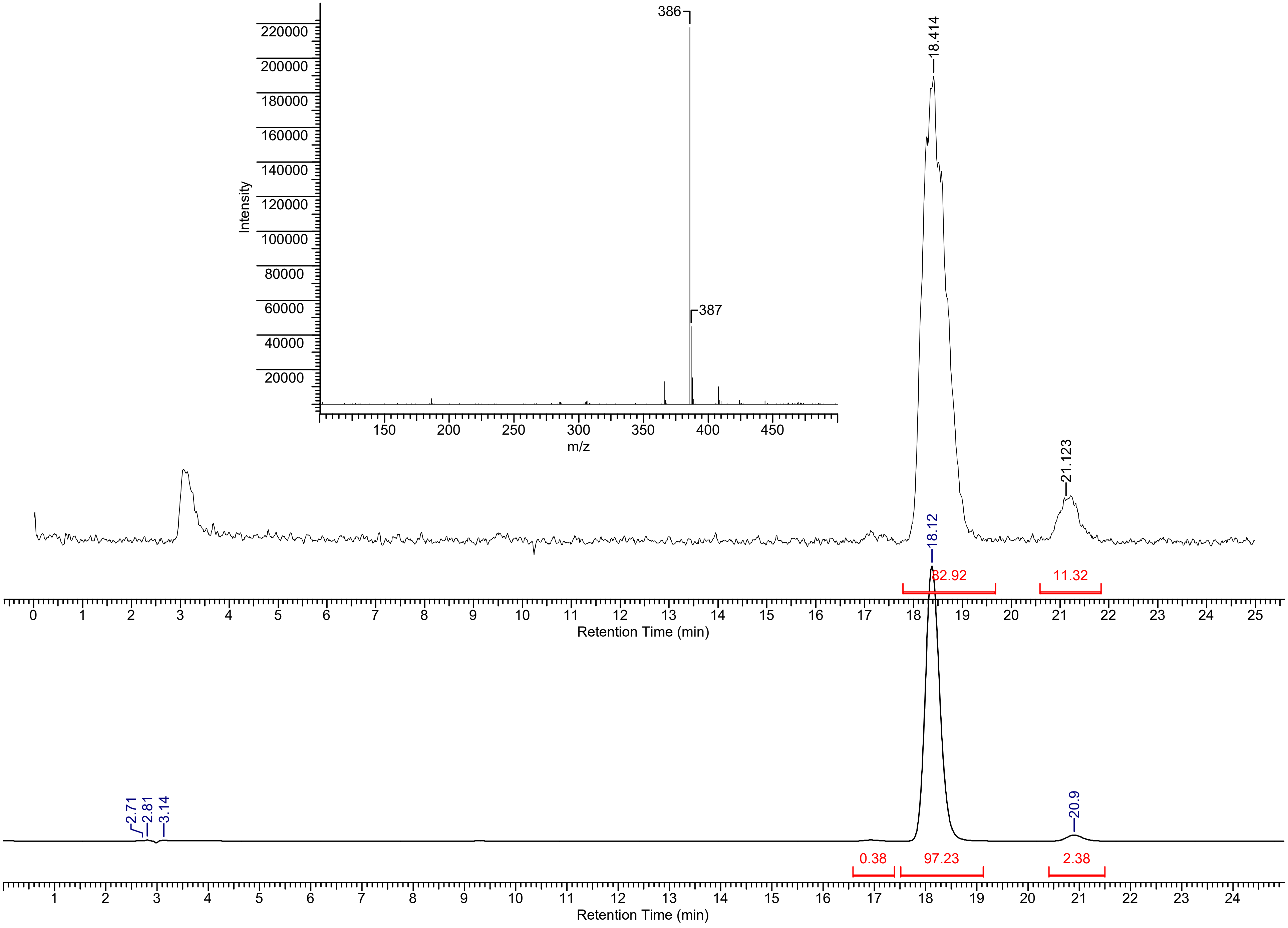

Supplement: S1 Spectra — (PDF) [file pone.0176606.s006.pdf]
